# Anti‐Motion Artifacts Iontronic Sensor for Long‐Term Accurate Fingertip Pulse Monitoring

**DOI:** 10.1002/advs.202414425

**Published:** 2025-02-22

**Authors:** Jia You, Mingyang Lu, Lamu Dazhen, Mengjie Gao, Ruiyan Zhang, Wendong Li, Fan Lei, Wei Ren, Guangxian Li, Junlong Yang

**Affiliations:** ^1^ College of Polymer Science and Engineering National Key Laboratory of Advanced Polymer Materials Sichuan University Chengdu Sichuan 610065 China; ^2^ Key Laboratory of Photovoltaic Materials School of Materials and New Energy Ningxia University Yinchuan Ningxia 750021 China; ^3^ School of Aeronautics and Astronautics State Key Laboratory of Polymer Materials Engineering of China Robotic Satellite Key Laboratory of Sichuan Province Sichuan University Chengdu Sichuan 610065 China; ^4^ School of Mechanical Engineering Chengdu University Chengdu Sichuan 610106 China

**Keywords:** anti‐motion artifacts, fingertip pulse monitoring, long‐term stability, soft‐hard stretchable interface, S‐smooth sensor

## Abstract

Flexible pressure sensors have gained attention for their comfort, portability, and potential in long‐term pulse monitoring and early cardiovascular disease diagnosis. However, stretching stress during daily activities affects sensor accuracy, causing motion artifacts (MAs) that hinder precise pulse signal detection. To address this challenge, the anti‐motion artifact iontronic pressure sensor (S‐smooth sensor), featuring a soft‐hard stretchable interface with energy dissipation properties is developed. By regulating the local modulus of the encapsulation layer, this structure dissipates stretching stress, achieving an MAs suppression rate of up to 90%, significantly improving pulse signal accuracy and reliability. Additionally, the sensor incorporates a dielectric layer and double electrode layer (EDL) sensing interface, with a low‐friction design that ensures high sensitivity (92.76 kPa−¹) and stability, maintaining performance over millions of cycles. The sensor accurately captures heart rate (HR) and pulse peak time differences (Δt) under various finger‐bending conditions. When integrated into a portable wireless pulse monitoring system, it shows a heart rate loss rate of only 2.9% during intense physical activity. This approach avoids complex chemical processes and material restrictions, offering a novel solution for motion artifact suppression in sensors, with significant potential for real‐time health monitoring and assisted diagnosis.

## Graphical abstract

Flexible pressure sensors are essential for pulse monitoring and cardiovascular disease diagnosis but are affected by motion artifacts (MAs) due to stretching stress. The S‐smooth sensor with a soft‐hard stretchable interface that dissipates energy, reducing MAs by 90% is developed. The sensor ensures high sensitivity (92.76 kPa^−^¹) and stability for one million cycles, providing a reliable solution for real‐time health monitoring, even during intense physical activity.

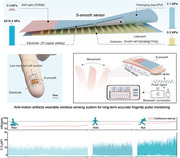

## 1 Introduction

Owing to their low‐modulus material properties, exceptional wearability, and high portability, flexible pressure sensors have emerged as critical components in medical health monitoring systems, enabling continuous and noninvasive physiological parameter monitoring.^[^ ^1^, ^2^, ^3^, ^4^, ^5^, ^6^ ^]^ These sensors are particularly valuable in long‐term pulse monitoring, where they can accurately capture pulse waveforms that reflect cardiovascular health. These waveforms contain critical physiological information, such as heart rate variability and arterial stiffness data, which are essential for assessing cardiac function and for the early detection of cardiovascular diseases.^[^ ^7^, ^8^, ^9^ ^]^ The accurate and continuous monitoring of pulse waves is therefore essential for enhancing diagnostic accuracy and reducing the incidence and mortality associated with cardiovascular conditions.

However, in dynamic daily activities such as walking, running, or swinging, the periodic contraction and extension of muscles exert additional tensile stress on wearable devices. This stress, characterized by a significant normal component, can interfere with pressure signal sensing, leading to the generation of “motion artifacts.” These artifacts severely distort the true shape of the pulse waveform, increase data noise, and degrade signal quality. The situation becomes particularly critical when the frequency of motion is similar to the heart rate, as the amplitude of the motion artifacts (MAs) may far exceed that of the pulse wave itself, leading to the complete masking of the pulse signal. This distortion of heart rate monitoring results can significantly compromise the accuracy of medical diagnostics, greatly increasing the risk of misdiagnosis or missed diagnosis.

The primary factors contributing to the generation of MAs in wearable device signals are the additional tensile stress and its normal components experienced during operation. To mitigate this issue, researchers have developed flexible electronic devices that minimize MAs through dual optimization of both material and structural design. On the material side, advances have been made in the development of conductive materials that maintain stable electrical performance under tensile strain,^[^ ^10^, ^11^, ^12^ ^]^ forming the basis for creating strain‐insensitive sensors. Structurally, the incorporation of foldable designs on the basis of Kirigami,^[^ ^13^, ^14^ ^]^ non‐planar structures,^[^ ^15^, ^16^ ^]^ and serpentine shapes^[^ ^17^, ^18^ ^]^not only provides high tensile properties but also minimizes normal deformation during stretching. This dual approach helps suppress MAs and ensures more stable sensing signals. The complex physical configurations of these foldable structures can compromise long‐term stability, as repeated deformation may lead to structural damage, thereby limiting their applicability in long‐term pulse monitoring. Additionally, the use of high‐modulus organic materials as isolation layers can effectively reduce the impact of MAs through strain dissipation mechanisms. For instance, microelectrodes made of epoxy resin^[^ ^19^ ^]^ and hard elastic island structures^[^ ^20^, ^21^ ^]^ constructed with high‐modulus materials have demonstrated good strain resistance while preserving structural stability. However, these high‐modulus materials also restrict the transmission of pulse vibrations, resulting in reduced sensor sensitivity. Consequently, the inherent trade‐off between high sensitivity and long‐term stability in the current strain‐insensitive sensors remains the key bottleneck hindering their widespread application in achieving continuous and reliable pulse monitoring.

Iontronic capacitive sensors have recently gained attention as a promising new direction in pressure sensor development, largely due to their exceptional sensitivity and tunable structure design.^[^ ^22^, ^23^, ^24^ ^]^ The heightened sensitivity is attributed to the formation of electric double layers (EDL) at the interfaces of ion‐conducting materials, where nanoscale charge separation occurs during sensing. These EDL can achieve ultrahigh specific capacitance, reaching several microfarads per square centimeter (µF cm^−2^) within the sub‐MHz frequency range.^[^ ^22^, ^25^ ^]^ Owing to their strong signal response, the electrode configuration can be shifted from a traditional sandwich structure to a coplanar arrangement without compromising sensitivity. This reconfiguration not only enables enhanced circuit integration but also reduces the misalignment between the dielectric layer and electrodes in 3D space, offering a potential solution to the issue of signal instability caused by deformation under complex stress conditions.

Here, we design a strain‐insensitive ionic capacitive pressure sensor (S‐smooth sensor) that features a slidable soft‐hard stretchable interface structure. This design effectively balances high sensitivity with superior resistance to MAs. Through the implementation of a coplanar electrode configuration, the construction of an iontronic sensing interface, and the meticulous optimization of the dielectric and electrode layer moduli, the sensor achieves an impressive sensitivity of 92.76 kPa^−1^. This high sensitivity enables the precise detection of pulse waveforms, facilitating the accurate measurement of heart rate (HR) and the subtle time difference (Δt) between the impact wave and tidal wave in pulse waveforms. To mitigate the impact of tensile stress, we introduce a low‐modulus polydimethylsiloxane (PDMS) soft segment at the stretchable end of the encapsulation layer, which results in the formation of a concentrated deformation zone. This strategy significantly reduces the normal stress during stretching, effectively suppressing up to 90% of MAs. Furthermore, by reducing the friction coefficient at the interface between the dielectric and electrode layers, we successfully prevent microstructure damage to the dielectric layer over 1 million cycles. Additionally, we develop a wireless pulse signal acquisition system (S‐smooth system) capable of continuously and accurately capturing pulse signals in various scenarios, spanning both static and dynamic conditions. The signal loss rate during running scenarios was dramatically reduced from 50% to 2.9% with the proposed sensor compared to that for sensors lacking MAs‐resistant structural designs. This advancement holds significant potential for applications in remote high‐quality medical monitoring, early disease diagnosis, and postoperative rehabilitation, providing a novel approach for the development of stretchable, highly sensitive, and stable electronic skin sensors.

## 2 Results

### 2.1 Design Principle of S‐Smooth Sensor

**Figure 1:**
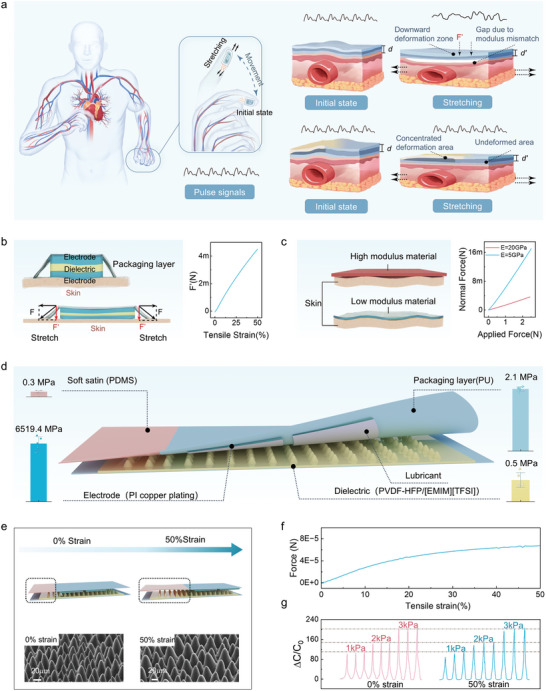
Design of anti‐motion artifacts S‐smooth sensor. a) Intended effects of rational and suboptimal structural designs on fingertip pulse signal monitoring. The strain‐sensitive design reduces signal errors caused by fingertip flexing during motion by minimizing stretching force (*F’*) and gaps, thereby enhancing signal stability compared to conventional flexible electronics. b) MAs arise from normal stress induced by tensile forces. c) MAs are caused by the inability of a high‐modulus substrate to efficiently transfer force. d) Schematic of the S‐smooth structure and mechanical modulus distribution of its constituent materials. e) Deformation in the strain concentration region and SEM images of the dielectric layer microstructure under 0% and 50% strain in the S‐smooth sensor. f) FEA results show variations in tip stress in the dielectric layer under strain. g) Capacitance changes under 1, 2, and 3 kPa pressure conditions at 0% and 50% strain.

The fingertip pulse is caused by periodic changes in the vascular walls due to the beating of the heart. Compared with other parts of the human body, the fingertips have an abundance of capillaries, making them more capable of accurately reflecting pulse waveforms.^[^ ^7^ ^]^ When a flexible pressure sensor is attached to a fingertip, the pressure changes at the fingertip are transmitted to the sensor through minor deformations, causing the dielectric layer to deform and thereby inducing signal changes.^[^ ^26^, ^27^ ^]^ However, the current wearable devices for pulse detection inevitably suffer from MAs,^[^ ^28^, ^29^, ^30^, ^31^ ^]^ especially in fingertip monitoring systems (**Figure** **1**a, left). This is because the tensile strain can lead to lateral contraction of the device, generating normal stress (*F’*) (Figure 1a, upper right). Finite element analysis (FEA) revealed that the normal stress *F*’ increases with the degree of skin stretching, causing additional signal changes triggered by nonpulsatile vibrations (Figures 1b and S1a, Supporting Information). Moreover, when there is a significant difference in the modulus between the substrate and the skin, gaps can form at the interface, hindering force transfer and leading to signal distortion (Figure 1a, upper right). The FEA results confirm that high‐modulus substrates are detrimental to force transfer (Figures 1c and S1b, Supporting Information), whereas low‐modulus materials that are compatible with the flexibility of the skin can effectively prevent gaps at the device‐skin interface during stretching.

Based on our design, we developed a strain‐insensitive sensor (S‐smooth), the structure of which is illustrated in Figure 1a–d. The sensor employs a highly flexible poly(vinylidene fluoride‐hexafluoropropylene) [P(VDF‐HFP)] polymer matrix, incorporating 1‐ethyl‐3‐methylimidazolium bis(trifluoromethylsulfonyl)imide [EMIM][TFSI] as the ionic liquid (IL). A microcone array ionic gel dielectric layer, prepared using a *Calathea zebrine* leaf as an inverse mold, provides both high sensitivity and outstanding uniformity (Figures S2 and S3, Supporting Information). Notably, this soft dielectric layer retains its microstructure even under 50% strain (Figure 1e) and exhibits a modulus comparable to human skin (0.5 MPa). To ensure seamless integration with the skin and minimize force loss, a coplanar electrode configuration (copper‐plated PI film) was selected, facilitating direct contact between the low‐modulus dielectric layer and the skin. This configuration eliminates gaps at the interface, enhancing the accuracy of pulse signal detection.

We further introduced an encapsulation structure aimed at reducing the *F’* generated during stretching. By applying 3 m gel to bond low‐modulus PDMS onto a section of the polyurethane (PU) encapsulation layer, we created a strain concentration region (Figure 1d). Lap shear tests show that the adhesion energy between PU and PDMS exceeds the fracture strength of PDMS (0.97 MPa), indicating a robust interface (Figure S4, Supporting Information). Since PDMS has a modulus of only one‐seventh that of PU, deformation is predominantly localized within the PDMS region during stretching (Figures 1e and S5, Supporting Information). This differential modulus design includes a sliding interface between sensor layers, thereby reducing the tensile stress within the encapsulation layer for a given deformation level, significantly mitigating the generation of *F’*. Additionally, conductive lubricating oil was applied to the electrode surface to lower the friction coefficient at the interfaces between device layers. This step further diminishes the effect of *F’* and safeguards the dielectric layer's structural integrity, thereby enhancing sensor stability. Consequently, we refer to this sensor as the “S‐smooth” sensor. The FEA results confirm that when the S‐smooth sensor undergoes large deformations, the stretching‐induced *F’* approaches zero (Figure 1f). This characteristic allows the sensor to maintain consistent signal output across various strain conditions, demonstrating exceptional strain insensitivity (Figures 1g and S6, Supporting Information).

### 2.2 Structural Regulation for Enhanced Sensitivity

**Figure 2:**
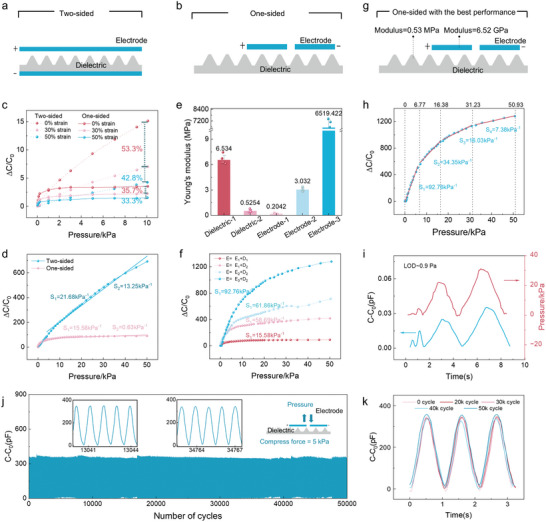
Optimized sensor design for high sensitivity. a) Schematic of the traditional sandwich structure (Two‐sided). b) Schematic of the coplanar electrode configuration (One‐sided). c) Normalized capacitance changes under pressure for sensors with One‐sided and Two‐sided structures across 0%–50% strain. d) Sensitivity comparison between One‐sided and Two‐sided sensor structures. e) Moduli of different electrode and dielectric layer materials. f) Normalized capacitance changes under pressure for sensors with different electrode and dielectric layer combinations. g) Schematic of the optimized sensor structure and materials system (S‐smooth sensor) with an electrode modulus of 6.5 GPa and a dielectric layer modulus of 0.5 MPa. h) Sensing sensitivity of the S‐smooth sensor. i) Limit of detection (LOD) of the S‐smooth sensor. j) Cyclic stability of the S‐smooth sensor over 50 000 cycles at a pressure of 5 kPa. k) Capacitive signals recorded at the first, 20 000th, 30 000th, 40 000th, and 50 000th compression cycles.

Sensitivity is a critical factor for evaluating sensor performance, and iontronic capacitive sensors achieve high sensitivity by forming a charge‐separated EDL interface at the nanoscale during sensing. To leverage this mechanism, the high‐conductivity ionic liquid [EMIM][TFSI] was incorporated into the soft P(VDF‐HFP) matrix, enabling the creation of microstructured dielectric layers for the construction of iontronic capacitive sensors. Our findings indicate that increasing the ionic liquid content in the dielectric layer gradually enhances the sensor's sensitivity (Figure S7, Supporting Information). However, excessive ionic liquid content raises the viscoelasticity of the ionic gel, which can compromise the stability of the dielectric layer (Figure S8, Supporting Information). To strike a balance between stability and sensitivity, the optimal ionic liquid content in the dielectric layer was determined to be 75 wt.%. Next, we discuss the structural design. The One‐sided electrode configuration has been demonstrated to exhibit superior strain‐insensitive in experiments.^[^ ^32^ ^]^ **Figure** **2** illustrates the conventional Two‐sided (sandwich) structure (Figure 2a), the One‐sided electrode configuration (Figure 2b), and the effects of different material moduli on sensor performance. The results show that, with the same low‐modulus electrode material (0.2 MPa), the initial capacitance amplitude changes in the One‐sided configuration under varying strain rates are significantly lower than those in the Two‐sided configuration (Figures 2c and S9, Supporting Information), with a 30% reduction in amplitude change (for a detailed explanation, see Section 2.3). However, the One‐sided configuration has the drawback of relatively lower sensitivity (Figure 2d). This reduction in sensitivity is attributed to the decreased contact area between the dielectric layer and the electrode in the One‐sided design, which weakens charge transport and subsequently lowers sensor sensitivity.^[^ ^33^, ^34^ ^]^

Adjusting the modulus can increase sensitivity. To verify this, we investigated the influence of varying the mechanical moduli of the electrode and dielectric layer materials on the sensor performance. By altering the dielectric layer matrix materials (PVDF and PVDF‐HFP) and electrode materials (fabric, PDMS coated with silver, and copper‐plated PI), we found that reducing the modulus of the dielectric layer while increasing the modulus of the electrode significantly improved the sensitivity of the one‐sided configuration, increasing it from 15.58 to 92.76 kPa^−1^, as shown in Figure 2e,f. This configuration also demonstrates excellent reproducibility (Figure S10, Supporting Information). This improvement is primarily due to the ease with which a low‐modulus dielectric layer deforms under applied force, and the complete transmission of this deformation to the high‐modulus electrode surface prevents strain loss during the process. As a result, the combination of a low‐modulus PVDF‐HFP dielectric layer (0.5 MPa) and a high‐modulus copper‐plated PI electrode (6.52 GPa) (Figure 2g) achieves highly sensitive monitoring (Figure 2h). This structure was subsequently employed in pulse monitoring studies.

Importantly, the optimized sensor substrate, with a modulus of 0.5 MPa, closely matches that of human skin, ensuring accurate transmission of pulse‐induced strain. Furthermore, the sensor has an ultralow limit of detection (LOD) of 0.9 Pa (Figure 2i) and a relaxation time significantly shorter than that of human skin (Figure S11, Supporting Information), which typically ranges from 30 to 50 ms.^[^ ^35^ ^]^ At a pressure of 1500 Pa, these flexible sensors maintain a high‐pressure resolution of 15 Pa or 1% (Figure S12, Supporting Information). This is due to the microstructure of the dielectric layer and the EDL sensing interface, which make the sensor highly sensitive to subtle mechanical stimuli while enabling rapid deformation and recovery. Finally, over 50 000 compression‐release cycles at 5 kPa, no appreciable signal drift was observed (Figure 2j,k), confirming the sensor's long‐term stability.

### 2.3 Optimization and Mechanism of Anti‐Motion Artifacts Performance in the S‐Smooth Sensor

**Figure 3:**
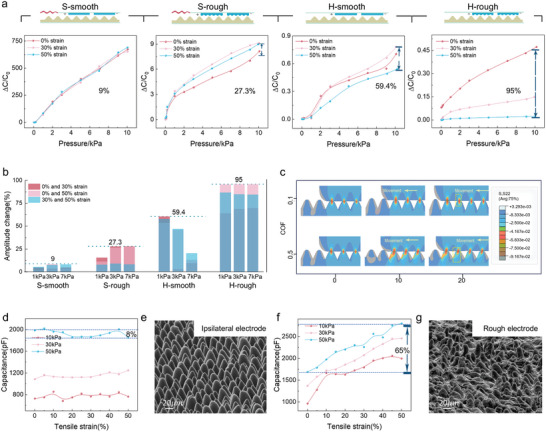
Optimized Sensor design for anti‐motion artifact performance. a) Pressure sensing performance of four structurally designed sensors under stretching conditions. b) Calculated capacitance amplitude changes of the four sensors when stretched under 1, 3, and 7 kPa pressures. c) FEA stress distribution of the electrode sliding on the dielectric layer with surface friction coefficients of 0.5 and 0.1 under normal pressure of 10 kPa. d,f) Capacitance signal changes of sensors with electrodes having friction coefficients of 0.1 (d) and 0.5 (f) when the dielectric layer is stretched to 50% strain under constant pressure. e,g) SEM images of the dielectric layer microstructure after 10 stretching cycles under 50 kPa pressure and 50% strain, in contact with electrodes having friction coefficients of 0.1 (e) and 0.5 (g).

To optimize the anti‐motion artifacts performance, we further assess the structural design of the S‐smooth sensor. The MAs in flexible pressure sensors are caused primarily by *F’* generated during stretching, which is closely related to the mechanical properties of the encapsulation layer and the friction coefficient at the stretched interface. To achieve superior anti‐motion artifacts performance, we designed four sensor structures by modifying the modulus gradient of the encapsulation layer, with PU as the stiff region and PDMS as the soft region to form a concentrated deformation zone, as well as by adjusting the surface roughness of the electrodes, as shown in **Figure** **3**a. The four sensors are characterized by i) soft‐hard encapsulation with a smooth electrode interface (S‐smooth), ii) soft‐hard encapsulation with a rough electrode interface (S‐rough), iii) hard encapsulation with a smooth electrode interface (H‐smooth), and iv) hard encapsulation with a rough electrode interface (H‐rough). We then examined their capacitive signal response under different strain conditions. To quantify their anti‐motion artifacts performance, we calculated the amplitude changes of the capacitive signal curves under various strains and pressures, as shown in Figure 3b (for calculation details, see Note S1, Supporting Information). The results indicate that the H‐rough sensor, which is composed of a hard PU encapsulation layer and rough electrode, exhibited the highest fluctuation of 95% in the pressure signal response under strain. However, when a PDMS low‐modulus region (0.3 MPa) was introduced at the front end of the encapsulation layer, the highest amplitude change decreased by 67.7%. This reduction suggests that the concentrated deformation zone at the front end effectively dissipates the high normal stress during the stretching process.

We further reduced the friction coefficient on the electrode surface by applying a thin lubricating layer to the rough electrode surface, which led to a further decrease in the normal stress. As shown in Figures S13 and S14 (Supporting Information), the lubricating layer reduced the electrode surface friction coefficient from 0.5 to ≈0.1. According to the results in Figure 3b, the amplitude changes in the H‐smooth sensor decreased from 95% to 59.4% compared with those in the H‐rough sensor, whereas those in the S‐smooth sensor decreased from 27.3% to 9%. This finding demonstrates that reducing the friction coefficient to ≈0.1 significantly enhances anti‐motion artifact performance. The S‐smooth sensor with both the soft‐hard modulus gradient in the encapsulation layer and the sliding interface design achieves only 9% amplitude fluctuation, indicating that the sensor successfully eliminates 90% of MAs and exhibits excellent anti‐motion artifacts performance. We compared the performance of the S‐smooth and S‐rough sensors in terms of capacitive signal variations under constant pressure and varying tensile strain, as shown in Figure 3d–f. The results revealed that regardless of whether the pressure was low (10 kPa) or high (50 kPa), the capacitive signal fluctuation was only 8% for the S‐smooth sensor. In contrast, the signal fluctuation was significantly greater (65%), confirming that the low‐friction sliding interface contributes to superior anti‐motion artifact performance.

Notably, the low friction coefficient also helps preserve the integrity of the microstructure of the dielectric layer. Figure 3e–g presents SEM images of the dielectric layer microstructure after ten tensile cycles under 50 kPa pressure and 50% strain for both the S‐smooth and S‐rough sensors. The S‐smooth sensor, with its low‐friction interface, shows minimal changes in microstructure after stretching, whereas the dielectric layer in the S‐rough sensor exhibits substantial bending and damage. This suggests that the introduction of a lubricating interface effectively protects the morphology of the dielectric layer. We attribute this to the low‐friction interface, which distributes stress more evenly and prevents localized concentrations of forces that could lead to bending and damage. This explanation is supported by the FEA results in Figures 3c and S15 (Supporting Information). This structural protection lays the foundation for the sensor's long‐term operational stability, enabling precise pulse signal detection over extended periods.

**Figure 4:**
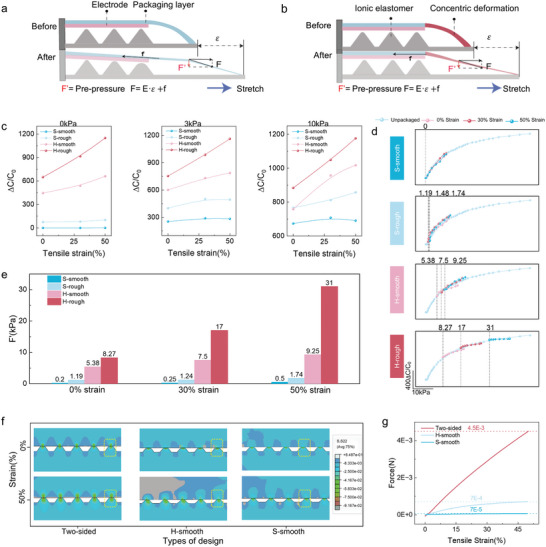
Mechanism of anti‐motion artifacts in the S‐smooth sensor. a,b) Schematic diagrams of stress analysis before and after stretching for traditional sensors (a) and sensors with soft‐hard encapsulation layers and sliding interfaces (b). c) Normalized capacitance signal changes in four structurally designed sensors under 0, 3, and 10 kPa pressures at 0–50% strain. d) Flowchart comparing data from sensors with different structural designs and unencapsulated sensors to determine *F'*. e) Comparison of *F'* caused by stretching in the four structural designs. f) FEA stress distribution at the microstructural tips of Two‐sided, H‐smooth, and S‐smooth sensors before and after 50% strain. g) Force exerted on the microstructural tips of the dielectric layer in sensors with different structures under 50% strain.

The primary mechanism associated with the strain insensitivity of the S‐smooth sensor is believed to be the reduction in *F'* generated during stretching. To confirm this, we conducted a force analysis of the sensor under strain. **Figure** **4**a shows the force distribution for a traditional sensor design, whereas Figure 4b presents the force distribution for the S‐smooth sensor. The mathematical model describing the tensile stress (*F*) on the encapsulation layer is as follows:

(1) F=E·ε+f

(2) f=μ·N

where *E* represents Young's modulus of the deformed section of the encapsulation layer, *ε* is the strain, *f* is the frictional force generated during stretching between the electrode and the dielectric layer, *μ* is the friction coefficient, and *N* is the normal force. As *μ* increases, the frictional force *f* also increases.

According to Equations (1) and (2), the additional *F’* that contributes to the MAs can be described as:

(3) $$F^{\prime }=F\cdot \sin\theta =\left(E\cdot \varepsilon +f\right)\times \sin\theta$$

where *θ* is the angle formed between the top encapsulation material and the substrate. Given that *θ* changes uniformly during stretching, we assume that sin*θ* is a constant to simplify the analysis. Thus, Equation (3) clearly shows that the sensor can reduce *F'* by lowering the modulus (*E*) and frictional force (*f*). Our S‐smooth sensor meets these criteria, demonstrating its ability to minimize *F'* and, consequently, MAs.

To quantitatively assess the impact of different structural designs on *F’* during stretching, the data from the four sensor configurations were normalized. This normalization was performed by using the initial capacitance (C_0_) of the unencapsulated sensor as a baseline and calculating ∆C/C_0_ to obtain the results shown in Figure S16 (Supporting Information). The purpose of this approach was to standardize all the data for comparison, minimize the influence of individual sensor performance variations, and provide an accurate reflection of changes in *F’*. The signals corresponding to 0, 3, and 10 kPa were extracted from Figure S16 (Supporting Information) and are presented in Figure 4c. The capacitance signal of the H‐rough sensor increased by ≈100% after 50% strain. However, after the concentrated deformation zone was introduced, the signal became significantly more stable, increasing by only 12% at 10 kPa under 50% strain. Finally, after the friction coefficient of the electrode was reduced, the signal change before and after stretching in the S‐smooth sensor was just 5%, confirming the effectiveness of the S‐smooth sensor in reducing *F’*. It is notable that the initial capacitance of sensors with different structures varies, mainly influenced by the PU hard‐encapsulation structure and friction coefficient between the electrode and dielectric layer (Figure S17, Supporting Information).

To further quantify the reduction in *F’* by the S‐smooth sensor, the processed data (Figure S16, Supporting Information) were incorporated into the capacitance‐pressure curve of the unencapsulated sensor. By horizontally shifting the normalized data along the *x*‐axis to align with the curve, the pressure corresponding to the initial capacitance was identified as the *F’* generated by structural stretching (Figure S18, Supporting Information). This process produced the results shown in Figure 4d. The comparative analysis of *F’* under different strains (Figure 4e) indicates that at 50 kPa, the induced soft‐hard strain concentration area corresponded to an *F*’ reduction from 31 to 1.74 kPa, resulting in a decrease of 94%. In contrast, the low‐friction interface aided in decreasing *F*’ from 31 to 9.25 kPa, achieving a reduction of 70%. For the S‐smooth sensor, the combined effect of these two characteristics led to the significant suppression of *F’*, which was reduced from 31 to 0.5 kPa, with a suppression rate of 98.3%. Furthermore, this suppression of *F’* became more pronounced as the strain increased. SEM observations of the microstructural changes after a single stretch cycle (Figure S19, Supporting Information) indicated that an increase in the friction coefficient did not lead to microstructural damage, suggesting that the primary role of the low‐friction interface is to reduce *F*’ rather than to protect the microstructure.

To further verify the accuracy of the data, we also conducted FEA to examine the stress (*F’*) at the tips of the microstructures in the dielectric layer for the two‐sided sensor, H‐smooth sensor, and S‐smooth sensor after applying a pre‐pressure of 1 kPa at 50% tensile strain. The results in Figure 4f indicate that compared with the traditional two‐sided electrode structure, the one‐sided electrode configuration significantly reduces the stress concentration at the tips of the microstructures. This reduction is likely due to the weaker deformation constraints of the two‐layer structure than those of the three‐layer structure. Moreover, the introduction of a low‐modulus region in the encapsulation layer further decreases *F’* to 7 × 10^−5^ for the S‐smooth sensor, representing a reduction of two orders of magnitude compared with that of the two‐sided sensor (Figure 4g). Importantly, for the sake of computational convergence, a damping coefficient of 0.0002 was applied in the H‐rough and S‐rough simulations. This resulted in the simulated contribution of the sliding interface being limited to only 3% (Figure S20, Supporting Information), although the results still aligned with the experimental trends. In conclusion, the FEA simulations were consistent with the experimental results, confirming that the soft‐hard modulus gradient design with a sliding interface effectively minimizes *F'* under strain, resulting in superior anti‐motion artifact performance.

### 2.4 Application of the S‐smooth Sensor for Long‐Term Precise Dynamic Pulse Monitoring

**Figure 5:**
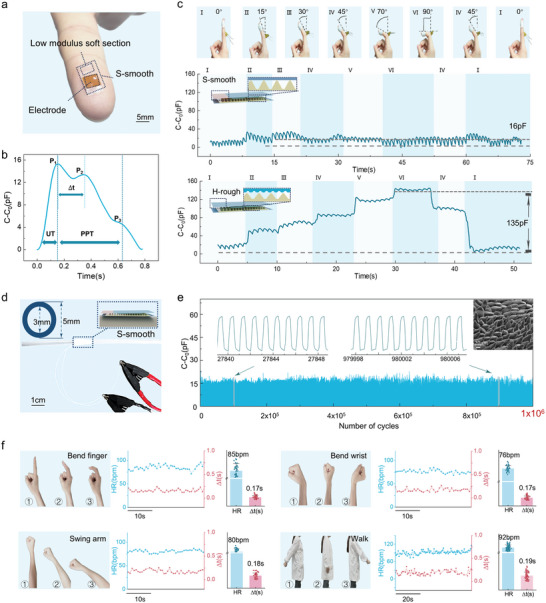
Application of the S‐smooth sensor in human pulse detection. a) Digital image of the S‐smooth sensor attached to a human fingertip. b) Capacitive signal changes of the fingertip pulse, displaying three distinct peaks. c) Fingertip pulse signals were obtained from the S‐smooth sensor and H‐rough sensor under different finger bending angles. d) Digital image of the test setup for stability testing of simulated pulse cycles using an air pump. e) Simulated pulse signal over 1 million cycles, with an inset SEM image of the dielectric layer microstructure after 1 million cyclic tests. f) Photos of pulse detection during four common daily activities using the S‐smooth sensor, along with the extracted HR and Δt.

A 5 mm × 10 mm S‐smooth sensor affixed to the fingertip of a healthy 24‐year‐old female volunteer was secured with medical tape (**Figure** **5**a) to monitor the pulse signals. The acquired pulse waveform is presented in Figure 5b. Owing to the high sensitivity of the S‐smooth sensor (92.76 kPa^−1^), we were able to capture precise pulse waveforms, which clearly exhibited characteristic percussion wave (P_1_), tidal wave (P_2_), and diastolic wave (P_3_). The sensor accurately measured the time interval (Δ*t*) between P_1_ and P_2_, with the volunteer's Δ*t* recorded at 0.18 s, falling within the normal physiological range.^[^ ^36^, ^37^ ^]^ Notably, Δ*t* serves as a vital physiological indicator reflecting arterial stiffness^[^ ^37^, ^38^, ^39^, ^40^, ^41^ ^]^ and is used in the diagnosis of conditions such as sinus arrhythmia.^[^ ^42^ ^]^ Thus, accurate detection of Δ*t* is crucial for cardiovascular disease diagnosis and health monitoring. Over the course of a week, multiple pulse measurements were conducted at regular intervals of 24 h for ≈5 s, yielding eight distinct pulse waveforms with clearly discernible Δ*t* values (Figure S21, Supporting Information), confirming the sensor's excellent stability and reproducibility.

To demonstrate the superior anti‐motion artifact performance of the S‐smooth sensor, both the S‐smooth sensor and H‐rough sensor were attached to the fingertip of the 24‐year‐old female volunteer to monitor pulse waveform signal changes under various finger bending conditions, as shown in Figure 5c and Movie S1 (Supporting Information). The fingertip was chosen for its broad range of motion‐bending the finger to 90° induces ≈40% strain, which prominently reveals MAs (Figure S22, Supporting Information). The results revealed that as the bending angle of the finger increased, the H‐rough sensor exhibited a noticeable baseline drift of ≈135 pF. This was particularly evident when the finger was bent to 90°, where the signal amplitude, which was influenced by MAs, was ten times greater than the actual pulse amplitude. In contrast, the S‐smooth sensor maintained a stable baseline (≈16 pF) under identical conditions, showing minimal influence from finger bending, thereby enabling accurate real‐time pulse detection even during dynamic motion, highlighting its exceptional anti‐motion artifact performance. It is noteworthy that fully encapsulating the S‐smooth sensor with PU causes 50% strain to induce a 240% signal fluctuation, thereby compromising strain insensitivity (Figures S23 and S24, Supporting Information). We attribute these increases in signal fluctuation and drift to reduced overall stretchability under a fully sealed design, which requires larger tensile forces at the same strain and thus induces a higher normal force (*F*’), ultimately amplifying motion artifacts.

Additionally, to verify the long‐term signal stability of the S‐smooth sensor in health monitoring applications, the sensor was affixed to a 3 mm silicone tube, which closely approximates the diameter of the radial artery.^[^ ^43^, ^44^, ^45^ ^]^ The experimental setup is shown in Figure 5d. Pulsation of the human artery was simulated via a pneumatic pump to cyclically inflate and deflate the tube, whereas the capacitance signal response was monitored in real‐time via an LCR bridge (Figure S25 and Movie S2, Supporting Information). Preload from the medical tape led to a high initial capacitance (C₀); during pump operation, vibrations caused stress relaxation at the bandage‐silicone interface, gradually reducing C₀ until it stabilized after roughly 800 s (Figures S25 and S26, Supporting Information). By adjusting the frequency and airflow of the pneumatic pump, artificial arterial waveforms mimicking the pulse rate (60 beats min^−1^) and amplitude (16 pF) of a human pulse were generated. As shown in Figure 5e, the S‐smooth sensor maintained excellent signal stability after 1 million cycles. SEM images of the dielectric layer's microstructure (Figure 5e, top right) revealed only minimal collapse and no structural damage, indicating remarkable durability. Given that a healthy adult heart typically beats 80 000–1 00 000 times per day, the S‐smooth sensor can provide stable, continuous pulse waveform monitoring for 8–10 days.

A key objective of long‐term pulse monitoring is to enable early disease diagnosis through the analysis of Δ*t* between specific waveform features. However, MAs can make it difficult to accurately determine Δ*t*. To demonstrate the potential of the S‐smooth sensor in early medical diagnostics, we evaluated its performance in monitoring pulse signals during four common human activities: finger bending, wrist bending, arm swinging, and walking. After the binding pre‐stress relaxed, the recorded pulse waveforms (Figure S27, Supporting Information) allowed extraction of heart rate (HR) and Δ*t* (Figure 5f). Owing to the high strain‐insensitive precision of the S‐smooth sensor, it was able to stably monitor an HR of ≈80 beats per min (bpm) and accurately identify Δ*t* values of ≈0.2 s. An elevated HR was also clearly observed during walking. Furthermore, the S‐smooth sensor's HR readings align closely with those from a commercial photoelectric pulse detection device (Figure S28, Supporting Information). Together, these results underscore the sensor's capability to reliably monitor both Δ*t* and HR even under various motion conditions, highlighting its potential for future clinical diagnostic applications.

**Figure 6:**
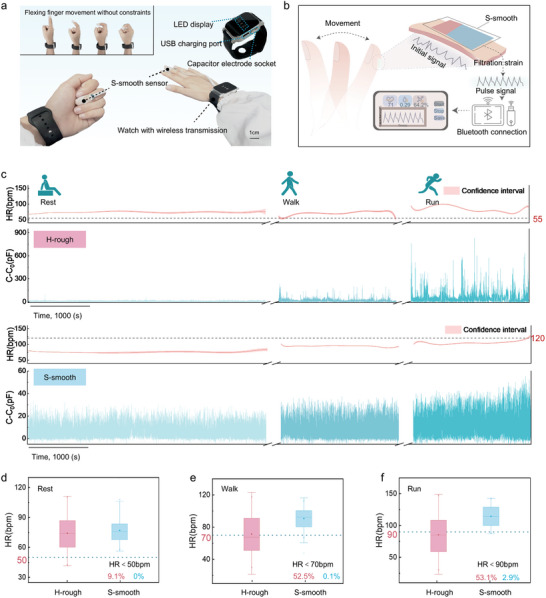
Application of the S‐smooth system for long‐term fingertip pulse monitoring. a) Digital images of the S‐smooth system, demonstrating its non‐restrictive wearing capability. b) Schematic of the working principle of the S‐smooth system. c) Fingertip pulse signals and HR data captured by the S‐smooth and H‐rough systems under resting, walking, and running conditions. d–f) Box plots showing HR measurements for both systems during rest (d), walking (e), and running (f), with the minimum, maximum, quartiles, and median values.

To enhance the portability of pulse waveform data collection and ensure long‐term comfort for the wearer, we developed a pulse detection system (S‐smooth system), as shown in **Figure** **6**a. The signal acquisition and transmission components were integrated onto a 3 cm × 3 cm circuit board, enclosed within a housing resembling a wearable watch, and equipped with a wristband for convenient wearing. The 5 mm × 10 mm S‐smooth sensor at the end was connected to the signal processing unit via a flexible circuit. During pulse monitoring, the user wore the data acquisition and processing unit on the wrist, with the S‐smooth sensor affixed to the fingertip and secured via medical tape. The S‐smooth system allows the user to move their fingers freely without interfering with daily activities, improving comfort for long‐term use. The collected pulse signals were transmitted via Bluetooth to a dedicated smartphone interface for real‐time monitoring and recording of physiological signals, as illustrated in the working mechanism diagram in Figure 6b. Importantly, the S‐smooth system only exports raw data. The HR and Δ*t* values presented in the figures were obtained through further data processing.

Figure 6c presents continuous pulse data collected over 8000 s via the S‐smooth system under resting, walking, and running conditions. For comparison, the S‐smooth sensor was replaced with the H‐rough sensor to capture fingertip pulse signals during the same activities. After further data processing, we defined the percentage of heart rates below 50, 70, and 90 bpm during resting, walking, and running, respectively, as the loss rate. The calculated heart rate loss rates for the S‐smooth and H‐rough sensors under the three conditions are shown in Figure 6d–f. A high loss rate indicates low HR accuracy and high susceptibility to MAs. The S‐smooth sensor system accurately measured heart rates of 80, 100, and 120 bpm during resting, walking, and running, respectively, with a loss rate of only 2.9% during running. In contrast, the H‐rough sensor exhibited loss rates of 52.5% and 53.1% during walking and running, respectively, indicating significantly inaccurate pulse signal acquisition. The lower loss rate of the S‐smooth sensor once again highlights its superior anti‐motion artifact performance, which is attributed primarily to its soft‐hard encapsulation layer design, which effectively eliminates the MAs caused by finger muscle stretching and arm swinging. Overall, the S‐smooth system effectively reduces MAs in HR monitoring, achieving an exceptionally low HR loss rate. Its portability, accuracy, and stability make it a highly promising long‐term pulse monitoring system for applications in fitness tracking, early medical diagnostics, and disease prevention.

## 3 Discussion

The MAs generated by flexible pressure sensors in dynamic environments can severely degrade signal quality, resulting in signal distortion and data errors. This interference is particularly critical in applications requiring high‐precision pulse detection. Eliminating MAs can significantly improve diagnostic accuracy in cases with medical technology and enable early disease detection. The two main causes of MAs are i) high‐modulus substrates that obstruct the accurate transmission of pulse forces and ii) *F’* induced by the dynamic stretching of the device. To address these issues, we developed an S‐smooth sensor, which features a sliding and soft‐hard stretchable interface. This design enables the accurate detection of fingertip pulse signals through a low‐modulus (0.5 MPa) and highly sensitive (92.76 kPa^−1^) dielectric layer. Additionally, the low‐friction soft‐hard interface reduces *F’* by more than 90%, effectively minimizing the occurrence of MAs. The sensor also demonstrates long‐term stability, withstanding over 1 million cycles of use without degradation of the sensing signal. We further developed a wireless, portable fingertip pulse monitoring system capable of long‐term, accurate fingertip pulse signal detection during dynamic human activities. Even during intense activities such as running, the system exhibited a heart rate loss of only 2.9%. This low loss rate, combined with the sensor's robust performance, highlights its significant potential for practical applications in cardiovascular disease diagnosis and health monitoring.

A comprehensive analysis of the underlying mechanisms revealed that stress dissipation is the key to reducing MAs. The primary mechanism of this design is the reduction in the localized modulus within the encapsulation layer, allowing for the dissipation of tensile stress and thereby achieving overall strain‐insensitive. The scientific significance of this approach lies in the localized softening encapsulation strategy and the monitoring of the deformation behavior of the differentially modulated encapsulation layer. This creates a sliding, soft‐hard, stretchable interface, effectively eliminating the normal pre‐stress caused by tensile deformation, which is common in flexible electronics. This effect was validated through data normalization and FEA. Compared with existing sensors,^[^ ^46^, ^47^, ^48^, ^49^ ^]^ the main advantage of the S‐smooth sensor is its combination of exceptional anti‐motion artifact performance, high sensitivity, and outstanding long‐term stability. As a result, it is capable of continuously and accurately monitoring pulse waveforms and extracting multiple physiological signals, including HR and Δ*t*, during dynamic human activities. This strategy avoids complex chemical processes and is not constrained by specific material or fabrication choices, making it adaptable for further applications in various dynamic monitoring scenarios, such as sports rehabilitation assessment, speech recognition, and human‐machine interaction. This design provides a theoretical framework for reducing MAs in flexible electronics, paving the way for the practical application of flexible electronic skins.

## 4 Conclusion

With a design strategy based on modulus optimization and interface regulation, we developed a flexible pressure sensor featuring a slidable soft‐hard stretchable interface, named S‐smooth. This sensor demonstrates robust resistance to motion interference and enables long‐term, accurate pulse monitoring. Notably, the integration of a soft‐hard modulus gradient within the encapsulation layer effectively reduces the normal stress induced by stretching, resulting in superior anti‐motion artifact performance, with up to 90% of MAs suppressed. The S‐smooth sensor also exhibits exceptional sensing characteristics, including high sensitivity (92.76 kPa^−1^) and durability, maintaining stable performance over 1 million cycles. These attributes allow the integration of S‐smooth into portable, miniaturized wireless pulse monitoring systems, achieving a heart rate loss of only 2.9%, even under strenuous conditions such as running, thus ensuring precise and reliable long‐term pulse monitoring. Given its high‐performance capabilities, the S‐smooth sensor is anticipated to have significant applications in early medical diagnostics, personalized healthcare, and postoperative rehabilitation.

## 5 Experimental Section

### Materials

Polydimethylsiloxane (PDMS, Dow Corning dc184) was purchased from Dow Corning Corporation. Poly(vinylidene fluoride‐hexafluoropropylene) [P(VDF‐HFP)], N, N‐dimethylformamide (DMF), and polyvinylidene fluoride (PVDF) were obtained from Aladdin. 1‐ethyl‐3‐methylimidazolium bis(trifluoromethylsulfonyl)imide ([EMIM][TFSI]) was procured from Lanzhou Institute of Chemical Physics, China. The polyurethane (PU) film dressing was sourced from Roldy Medical Devices. The conductive lubricant was purchased from Shenzhen EUBO New Materials Technology Company, and the main components were low viscosity polyalphaolefin (PAO) and conductive carbon black solids.

### Preparation of PDMS Template and Dielectric Layers

Fresh *Calathea zebrina* leaves were immersed in deionized water and ultrasonicated to remove impurities. Rectangular portions were cut from the central leaf regions for consistency in microstructure. The leaves were air‐cleaned, fixed onto glass, and coated with a 5:1 PDMS mixture. This coating was cured at room temperature for 12 h to yield a “negative” PDMS mold, which could be reused multiple times to replicate the leaf's microstructure in subsequent dielectric layers. For the dielectric layer, 2 g of PVDF‐HFP (Aladdin) was dispersed in 10 g of N, N‐dimethylformamide (DMF, Aladdin) at 80 °C for 2 h. Next, 6 g of [EMIM][TFSI] (Lanzhou Institute of Chemical Physics, China) was added, followed by stirring at room temperature for 2 h. The solution was coated onto the PDMS template and dried at 80 °C to form a 100 µm‐thick ion gel film. The negative microstructure of the PDMS template, mimicking velvet *Calathea zebrina* leaves, imparted micro‐pillar structures to the ion gel, serving as the dielectric layer.

### Preparation of the S‐Smooth Sensor

The S‐smooth sensor was constructed using copper‐plated PI as electrodes and PVDF‐HFP as the dielectric layer. A 100 µm‐thick PDMS film (Sylgard 184, 20:1 with curing agent) was used as the soft segment, while commercial PU dressing was employed as the hard segment. A conductive lubricant was applied to the electrodes via a scraper to reduce friction. Electrodes and the dielectric were attached to the hard PU segment using 3 m gel, forming an S‐smooth sensor with a sliding soft‐hard interface. The final sensor measured 10 mm × 28 mm, with a 10 mm × 10 mm sensing area matching the 10 mm × 10 mm soft segment. The electrodes measured 10 mm × 10 mm, while the dielectric was 10 mm × 15 mm. Hard segments for the electrodes and dielectric were 10 mm × 20 mm and 10 mm × 28 mm, respectively. The soft segment adhered 3 mm from the left side of the hard segment, aligning the left edges of the electrodes and dielectric.

### Sensing Performance of S‐Smooth Sensor

The effective sensing area of the sensor for performance evaluation was standardized to 10 mm × 10 mm. Sensitivity and detection thresholds were assessed by mounting the sensor onto the planar platen of a universal mechanical testing machine (XLD‐1000E, Guangzhou Jingkong Testing Inc.). A flat indenter, matching the sensor dimensions, was maneuvered to establish minimal contact. The loading program (XLD‐20E, King Kong Mechanical Testing Co., Ltd.) was initiated, and real‐time capacitive signals were captured using an LCR meter (TH2840A, Tonghui Inc.) at 10 kHz. To measure response and release times, a 500 gram mass was placed on the sensor's upper surface, exerting a pressure of 40 kPa. The mass was released after a dwell time of 10 s, and the capacitive signals were recorded with the LCR meter, allowing for precise determination of response and recovery times.

### Anti‐Motion Artifacts Performance

The sensor was clamped at both ends using a tensile platform. For the tests of S‐smooth and S‐rough sensors, a 50% strain in the soft segment was considered equivalent to a 50% overall elongation of the device. In the tests of H‐smooth and H‐rough sensors, a 50% deformation of the hard segment at the front end signified a 50% total elongation. The tensile platform was positioned on the flat surface of the mechanical tensile testing machine, and sensor performance was evaluated by adjusting the indenter position for minimal contact.

### Finite Element Analysis (FEA) of MA Generation Mechanisms

Quasi‐static FEA simulations were performed in Abaqus using an implicit algorithm. The moduli of the electrode and dielectric layers were set at 6.6 GPa and 0.4 MPa, respectively, with an interfacial friction coefficient of 0.5. First, a uniform load of 10 kPa was applied, followed by 50% stretching in the x‐direction, generating force at the dielectric layer tip. The effect of substrate modulus on pulse signal accuracy was investigated by varying the modulus (20 and 5 GPa). Upward displacement was imposed, and forces experienced by microstructures were analyzed.

### FEA for the Mechanism of Sliding Interface

The electrode layer modulus was set to 6.6 GPa, and the dielectric layer modulus to 0.4 MPa. A 10 kPa load was applied, followed by a 50% *x*‐direction stretch. Varying the interfacial friction coefficients to 0.1 and 0.5, horizontal friction force and contact area were analyzed to understand the influence of interfacial friction on capacitive signals.

### FEA for Anti‐Motion Artifacts Mechanism

FEA simulations were conducted to evaluate the strain‐insensitive mechanism. Using quasi‐static analysis in Abaqus, the moduli of materials and friction coefficients (0.1 and 0.5) matched experimental values. The *F*’ generated on the microstructured surface under 50% tensile strain was analyzed for various sensor designs, demonstrating that the S‐smooth sensor effectively reduces MA at its source.

### S‐Smooth Sensor Stability Test

The S‐smooth sensor (5 mm × 14 mm) was applied to a 3 mm inner diameter silicone tube. One end of the tube was sealed, while the other was connected to an air pump inflating and deflating the tube every second, simulating a human pulse. Capacitance signal changes were monitored using an LCR meter (TH2840A, Tonghui Inc.).

### Fingertip Pulse Detection under Dynamic Conditions

A scaled‐down S‐smooth sensor (5 mm × 14 mm) was specifically designed for fingertip pulse detection. To ensure stability during use, the sensor was secured to the index finger using specialized medical tape. Volunteers, having provided fully informed consent, wore the S‐smooth sensor for pulse detection experiments. After the preload‐induced relaxation reached equilibrium, pulse signals were monitored under various dynamic conditions such as finger bending, arm swinging, wrist bending, and walking. Capacitance data were acquired via the LCR meter and analyzed in MATLAB to extract heart rate (HR) and the time interval (Δ*t*). The S‐smooth sensor consistently maintained a stable fit on the fingertip during these activities, ensuring reliable data acquisition under dynamic conditions.

### Fingertip Pulse Monitoring of the S‐Smooth System and H‐Rough Systems under Dynamic Conditions

The S‐smooth system comprised a capacitive monitoring unit, storage unit, and Bluetooth transmission unit. Data were stored in a mobile application and filtered in MATLAB for HR calculation. The S‐smooth and H‐rough sensors (5 mm × 14 mm) were affixed to fingertips via flexible FPC cables. The sensors were subjected to 4000 s of resting, 2000 s of walking, and 2000 s of running tests.

### Characterization

The dielectric layer microstructure was characterized by field emission scanning electron microscopy (SEM, ZEISS Sigma 300). Young's modulus was tested using an intelligent tensile testing machine (XLD‐1000E) at a tensile rate of 10 mm min^−1^.

## Conflict of Interest

The authors declare no conflict of interest.

## Author Contributions

J.Y. proposed and supervised the project. J.Y., M.L., and J.Y. conceived and designed the experiments. J.Y., L.D., M.G., R.Z., and W.L. performed the experiments and analyzed the data. G.L., R.Z., F.L., and J.Y. gave guidance on the data analysis and paper revision. J.Y., W.L., M.L., W. R., and J.Y. wrote the manuscript.

## Supporting information



Supporting Information

Supplemental Movie 1

Supplemental Movie 2

## Data Availability

The data that support the findings of this study are available from the corresponding author upon reasonable request.
