# Supplementary material for: Anti‐Motion Artifacts Iontronic Sensor for Long‐Term Accurate Fingertip Pulse Monitoring
Source: Adv Sci (Weinh). 2025 Feb 22;12(15):2414425. doi: 10.1002/advs.202414425 (PMC12005763; doi:10.1002/advs.202414425)
Supplement: Supplementary file 1 — Supporting Information [file ADVS-12-2414425-s001.docx]

*Supplementary Information*

**Anti-Motion Artifacts Iontronic Sensor for Long-Term Accurate Fingertip Pulse Monitoring**

*Jia You, Mingyang Lu, Lamu DAZHEN, Mengjie Gao, Ruiyan Zhang, Wendong Li, Fan Lei, Wei Ren, Guangxian Li, Junlong Yang**

**Note S1 |** The calculating process of the amplitude change:

*Amplitude Change* $=\frac{\left| a-b \right|}{b}$

where a is the ΔC/C_0_ signal after stretching at a certain pressure, and b is the ΔC/C_0_ signal before stretching at the same pressure.


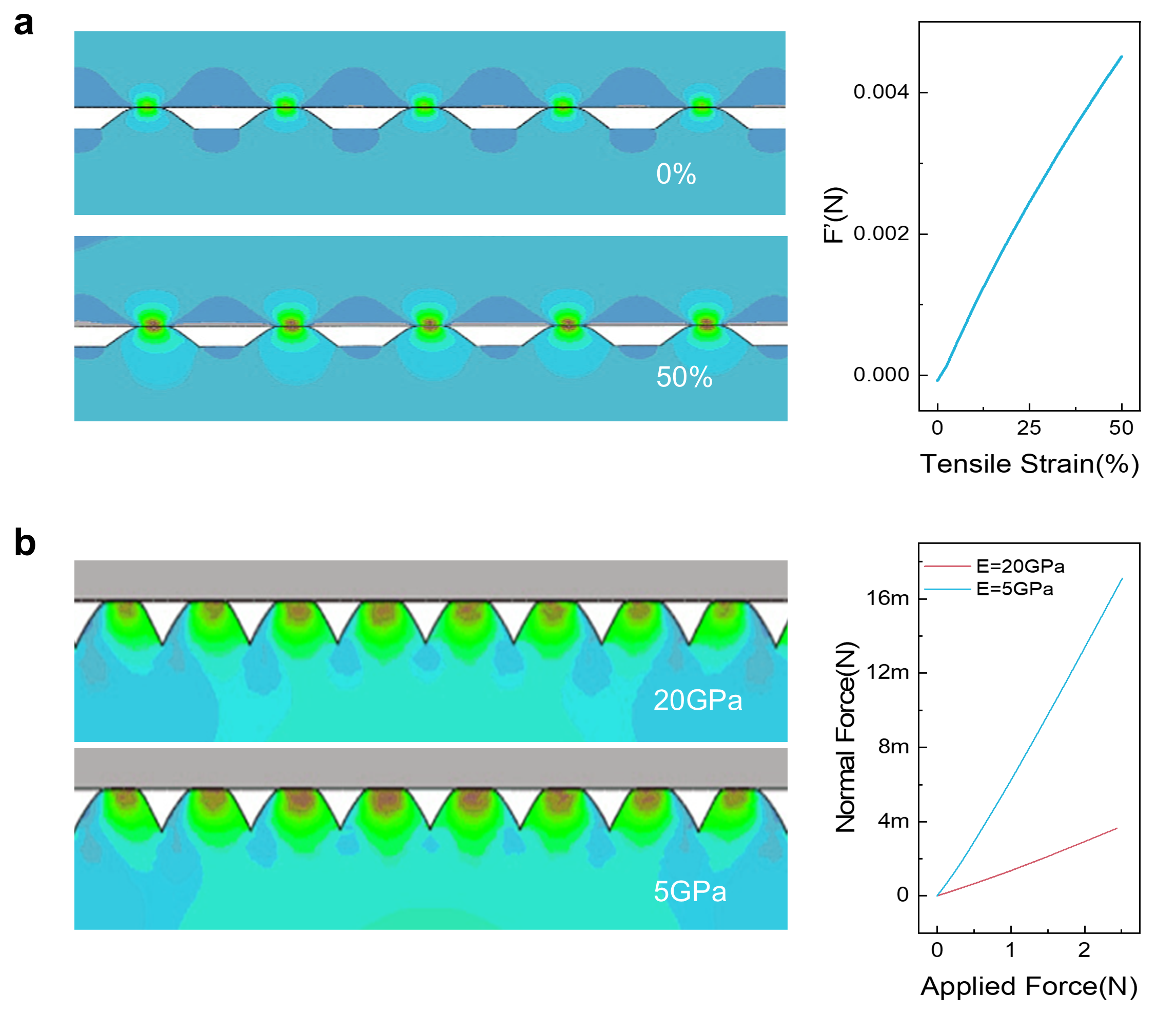


**Figure S1.** (a) Finite Element Analysis (FEA) stress distributions in the dielectric layer microstructure at 0% and 50% strain. (b) FEA stress distributions at the microstructure tip on substrates with 20 GPa and 5 GPa modulus under continuous stress application.


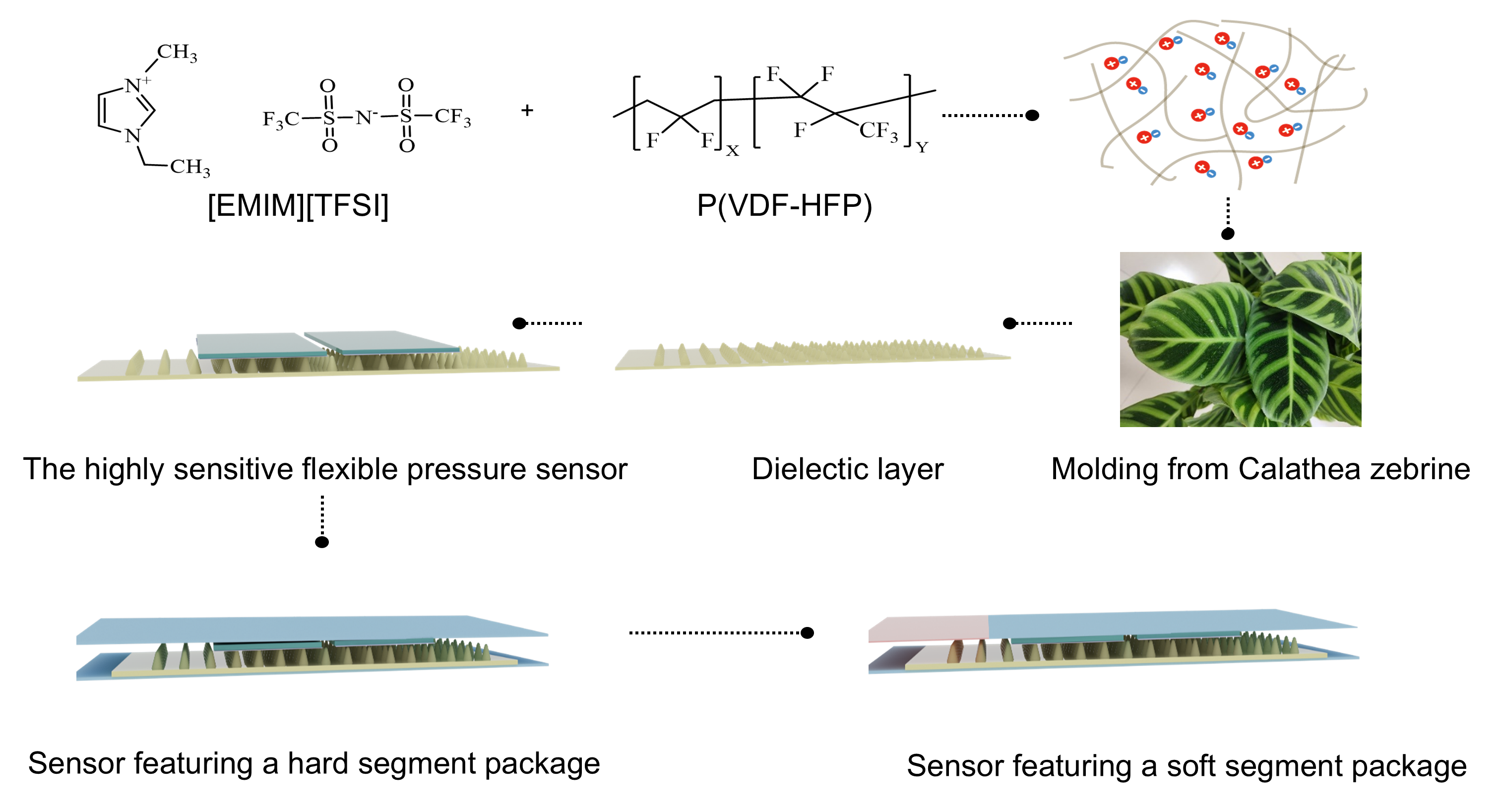


**Figure S2.** Fabrication process of the anti-motion artifacts iontronic pressure sensor (S-smooth sensor).

First, [EMIM][TFSI] was blended with P(VDF-HFP) and coated onto a PDMS mold with a *Calathea zebrina* leaf microstructure at 80°C for 4 hours to form an ionic gel dielectric layer. A PI copper-plated film served as the electrode. Using a co-planar electrode assembly configuration, the S-smooth sensor was constructed with a rigid PU encapsulation layer. Additionally, a PDMS soft segment was connected to the left end using 3M gel, creating a soft-to-hard modulus gradient to regulate strain concentration and deformation zones.


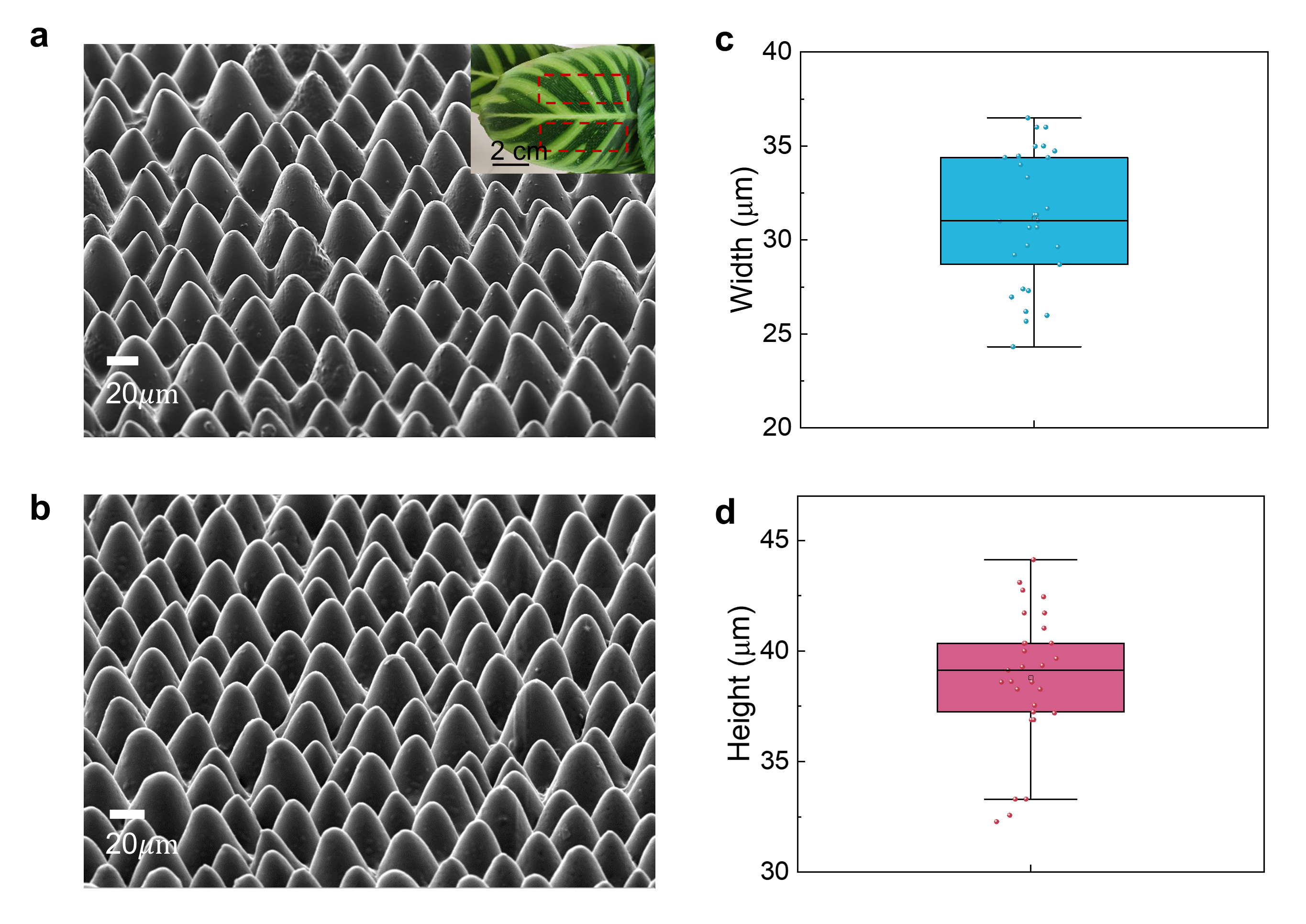


**Figure S3.** SEM images of dielectric layers prepared at different times. (a) The first fabricated dielectric layer, with the replicated leaf region shown in the top-right corner. (b) The second fabricated dielectric layer. (c) Average width of the ionic gel microstructure is 31.11 $\mu m$ with an error of 11.19%. (d) Average height of the ionic gel microstructure is 38.78 $\mu m$ with an error of 7.78%.The microstructure exhibits excellent consistency: there are approximately 1000 microcolumns per square millimeter, showing an error of 8%.


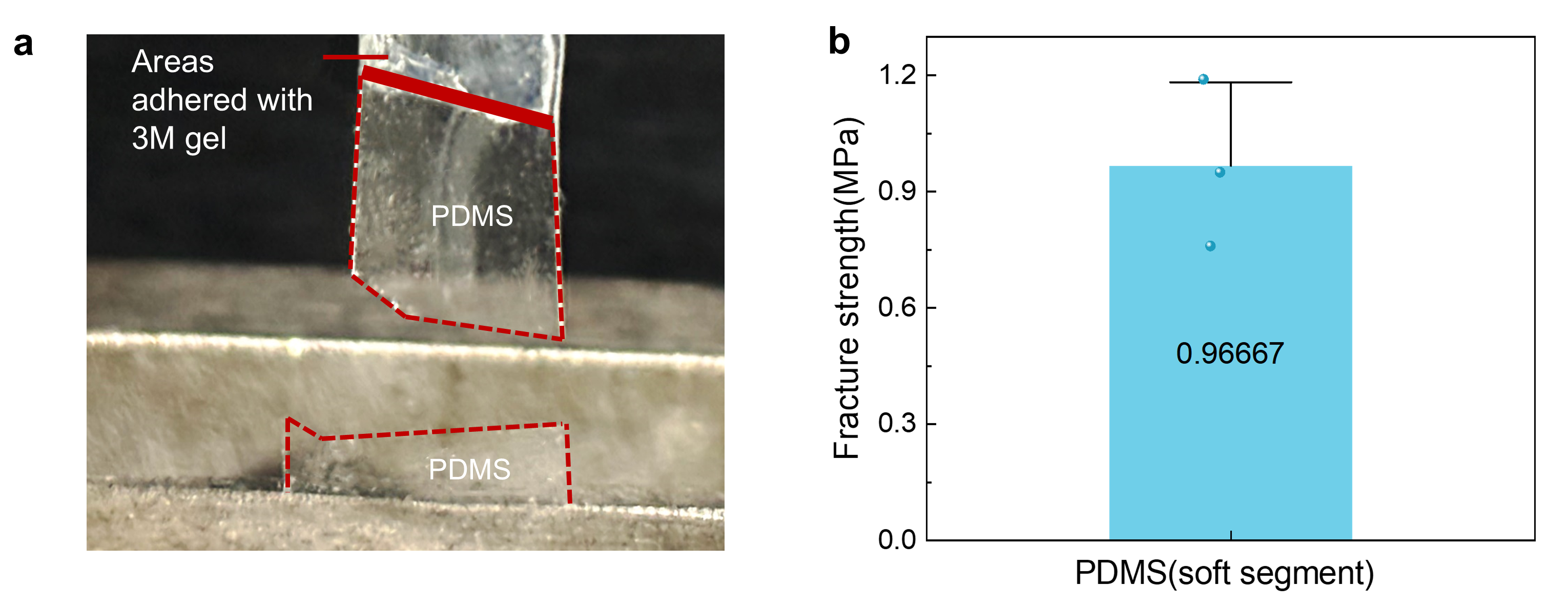


**Figure S4** (a) Photograph of fracture occurring in PDMS after lap shear testing. (b) Fracture strength of PDMS.


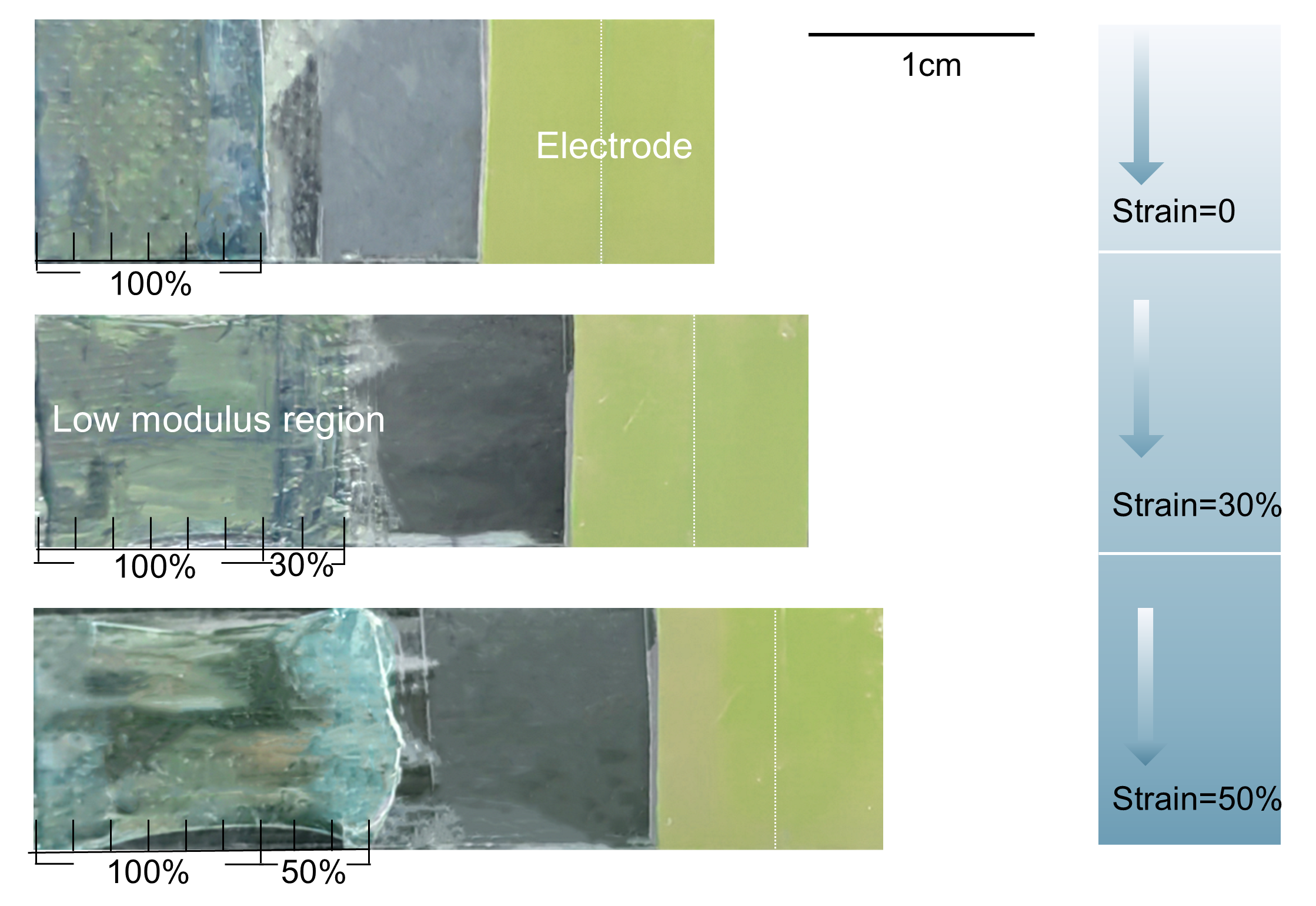


**Figure S5.** Digital images of the S-smooth sensor under different tensile strains, showing the low modulus soft section at the front end forming a concentrated deformation zone during stretching


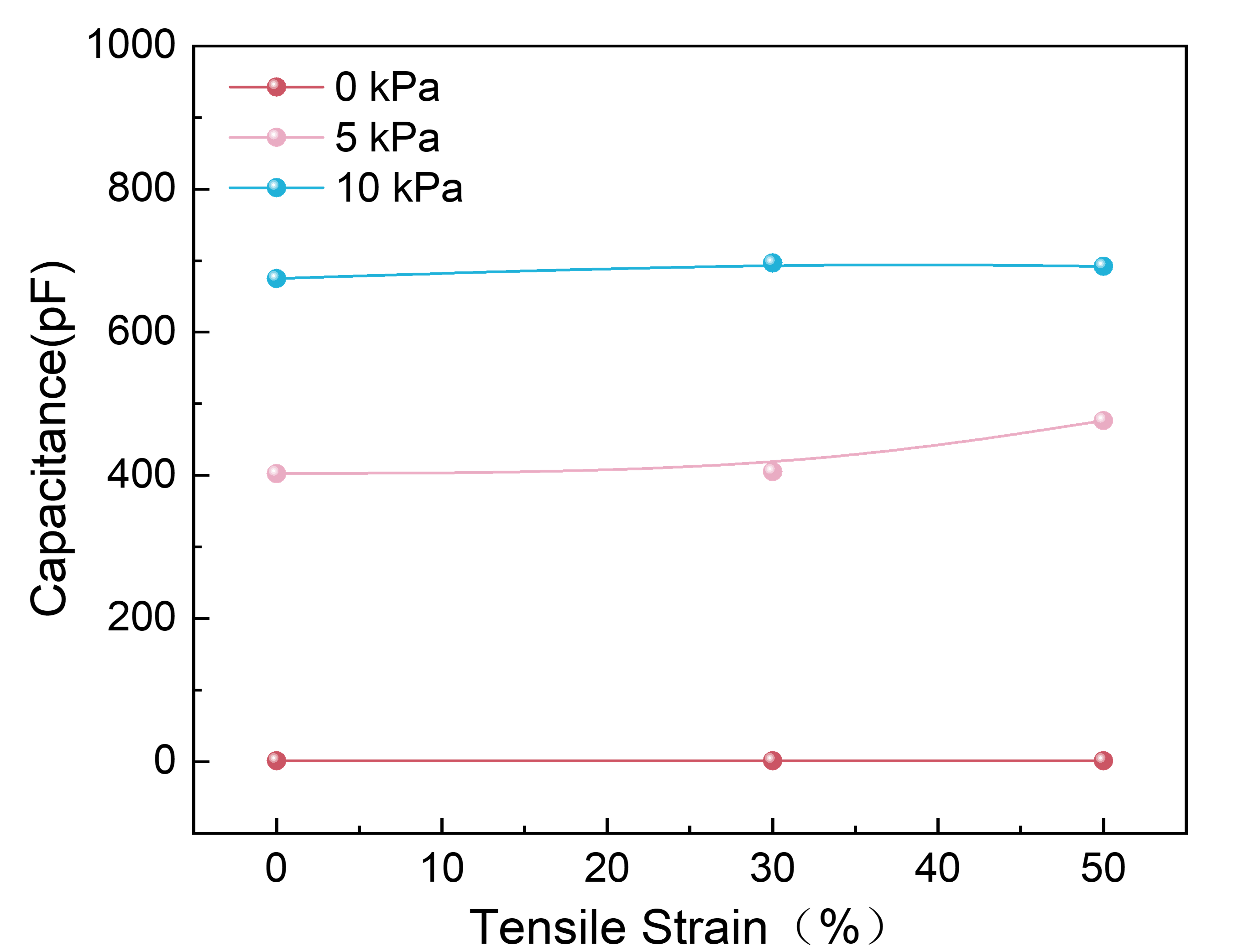


**Figure S6.** Strain-capacitance curves of S-smooth sensor from 0% to 50% at different pressure.


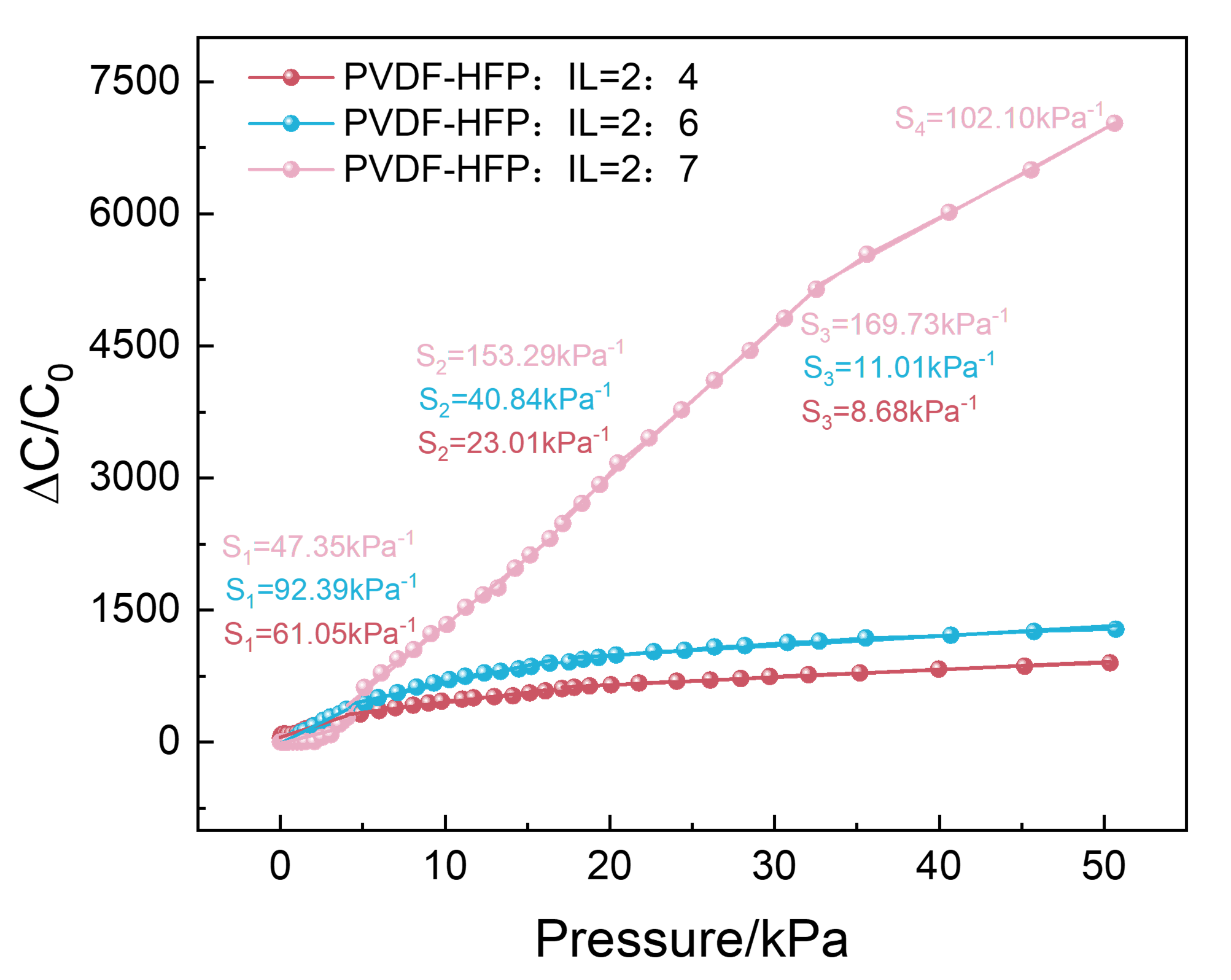


**Figure S7.** Sensitivity curves of sensors with dielectric layers containing different ionic liquid (IL) contents.


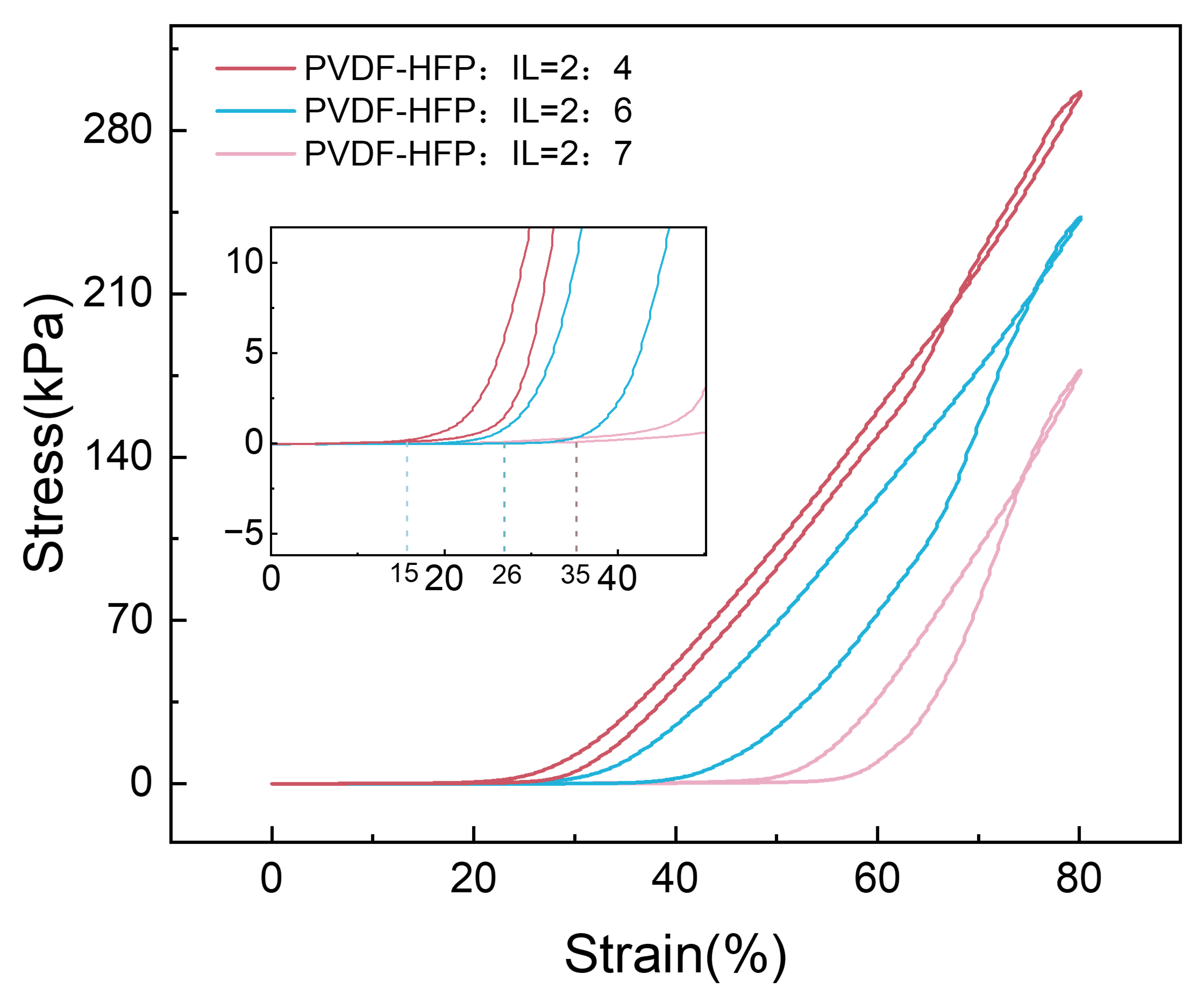


**Figure S8.** Cyclic stress-strain curves of PVDF-HFP/ionic liquid materials with different ionic liquid loadings. The displayed curve represents the seventh cycle from a series of seven cyclic tests. The results indicate that the viscoelasticity of the material increases with higher ionic liquid content, which is detrimental to the recovery of the dielectric layer.


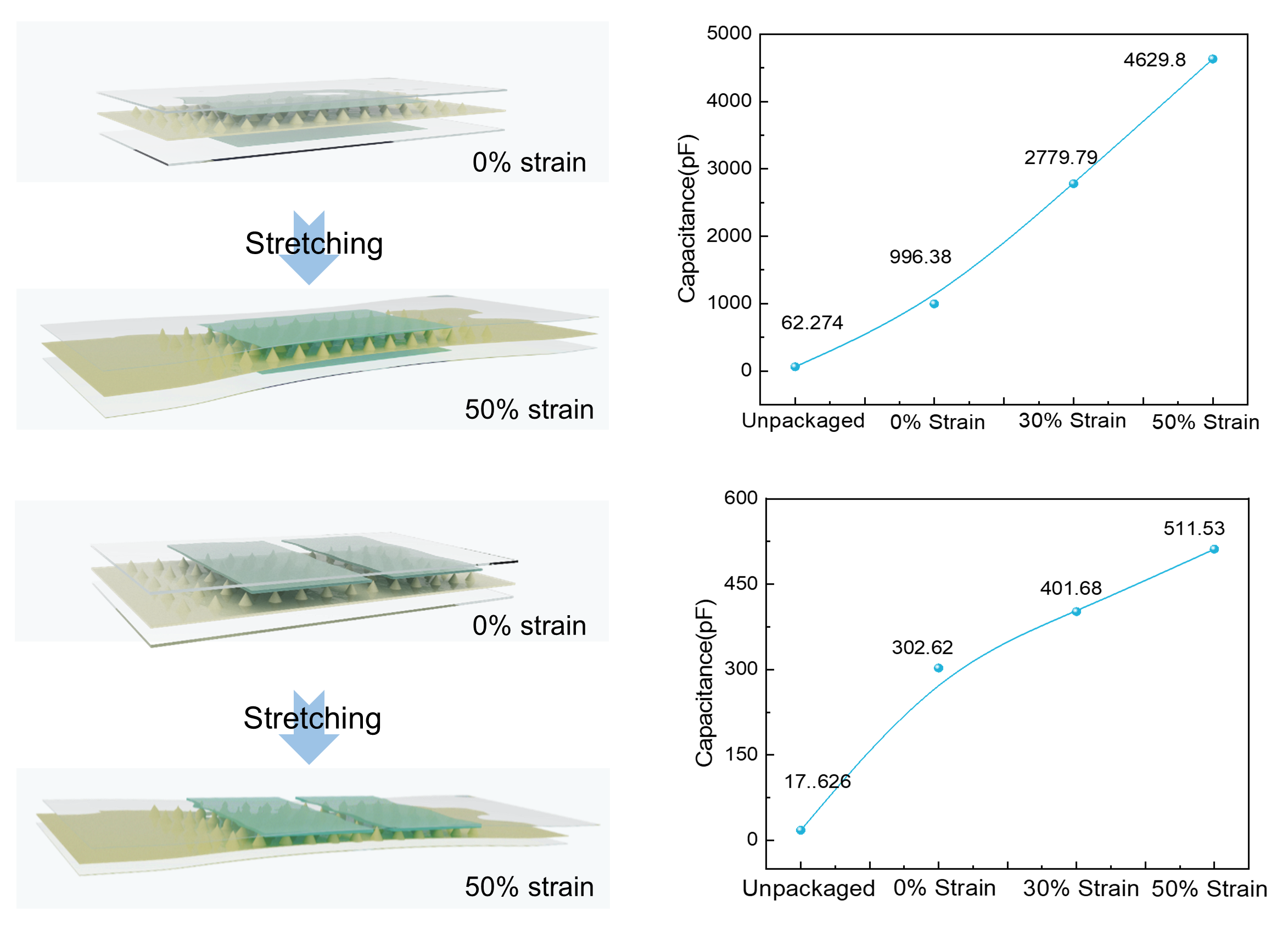


**Figure S9.** Initial capacitance signals under stretching for both the conventional sandwich structure (top and bottom electrodes with a middle dielectric layer) and the co-planar electrode structure. It should be noted that the modulus of the electrode used is 0.2 MPa. The initial capacitance of the conventional sandwich structure increases by 4.6 times when stretched to 50%, whereas the co-planar electrode structure shows a 1.7-fold increase under the same strain.


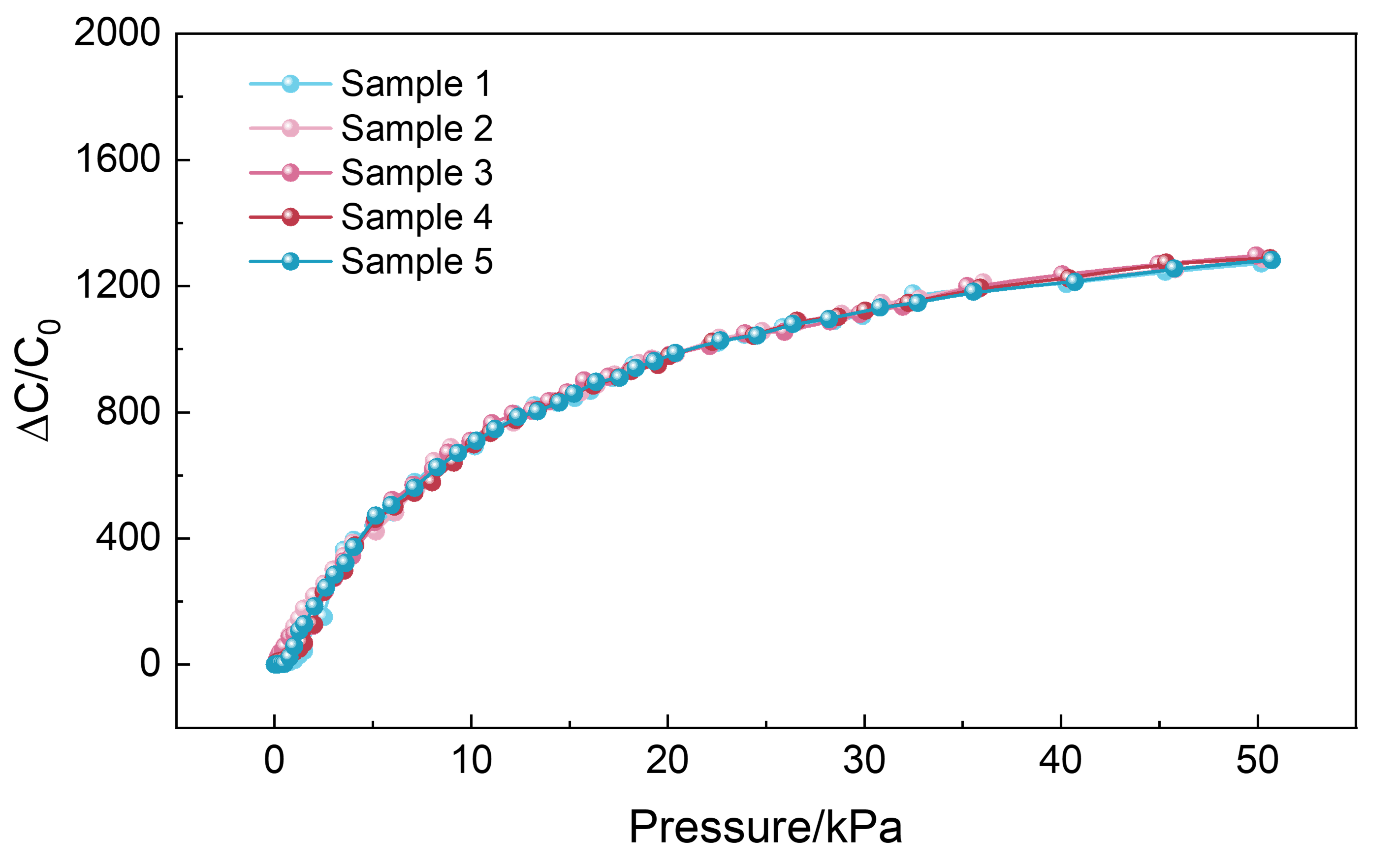


**Figure S10.** Sensitivity curves of 5 sensors from different batches, showing an excellent repeatability.


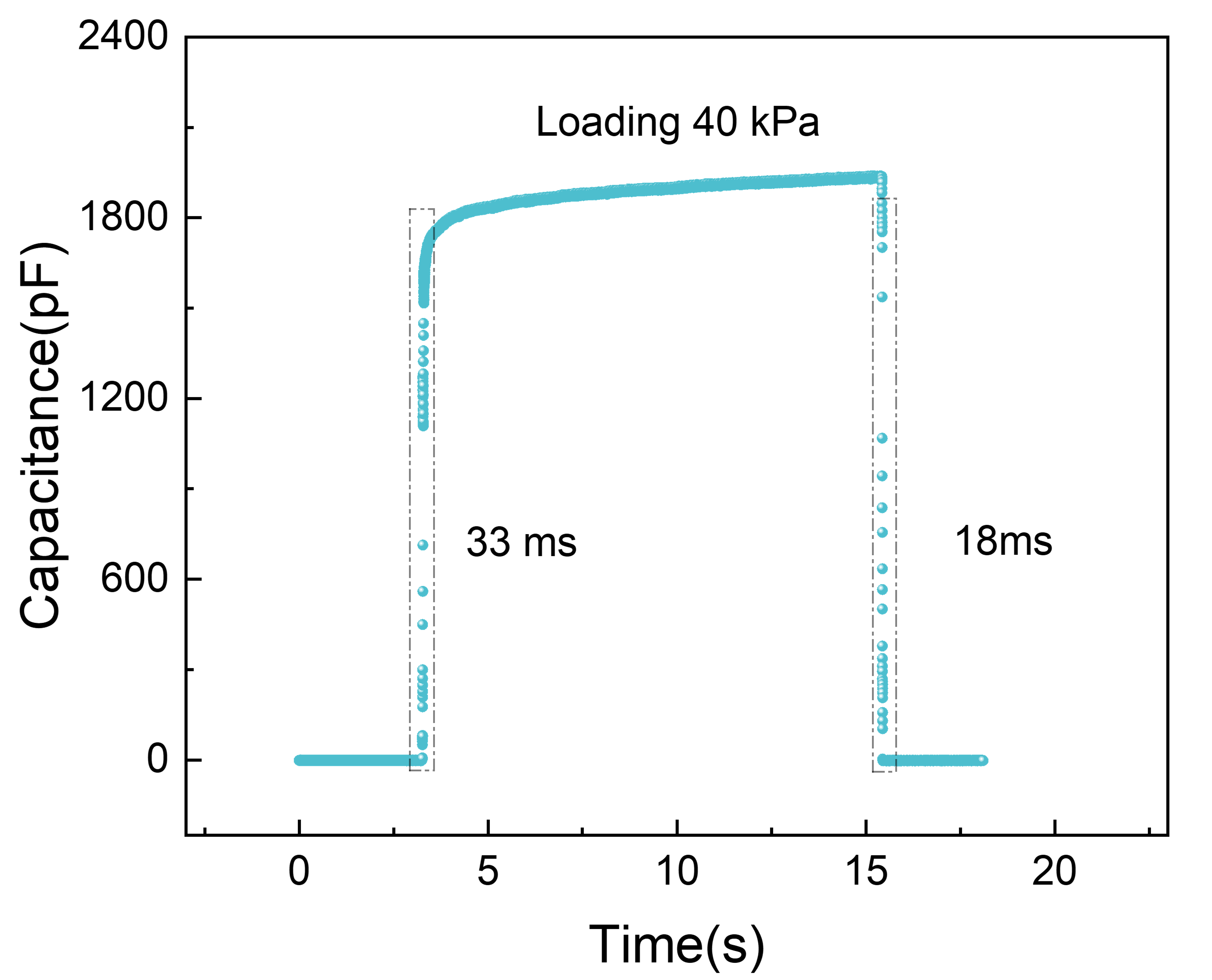


**Figure S11.** Response and recovery time of S-smooth sensor under a pressure of 40 kPa.


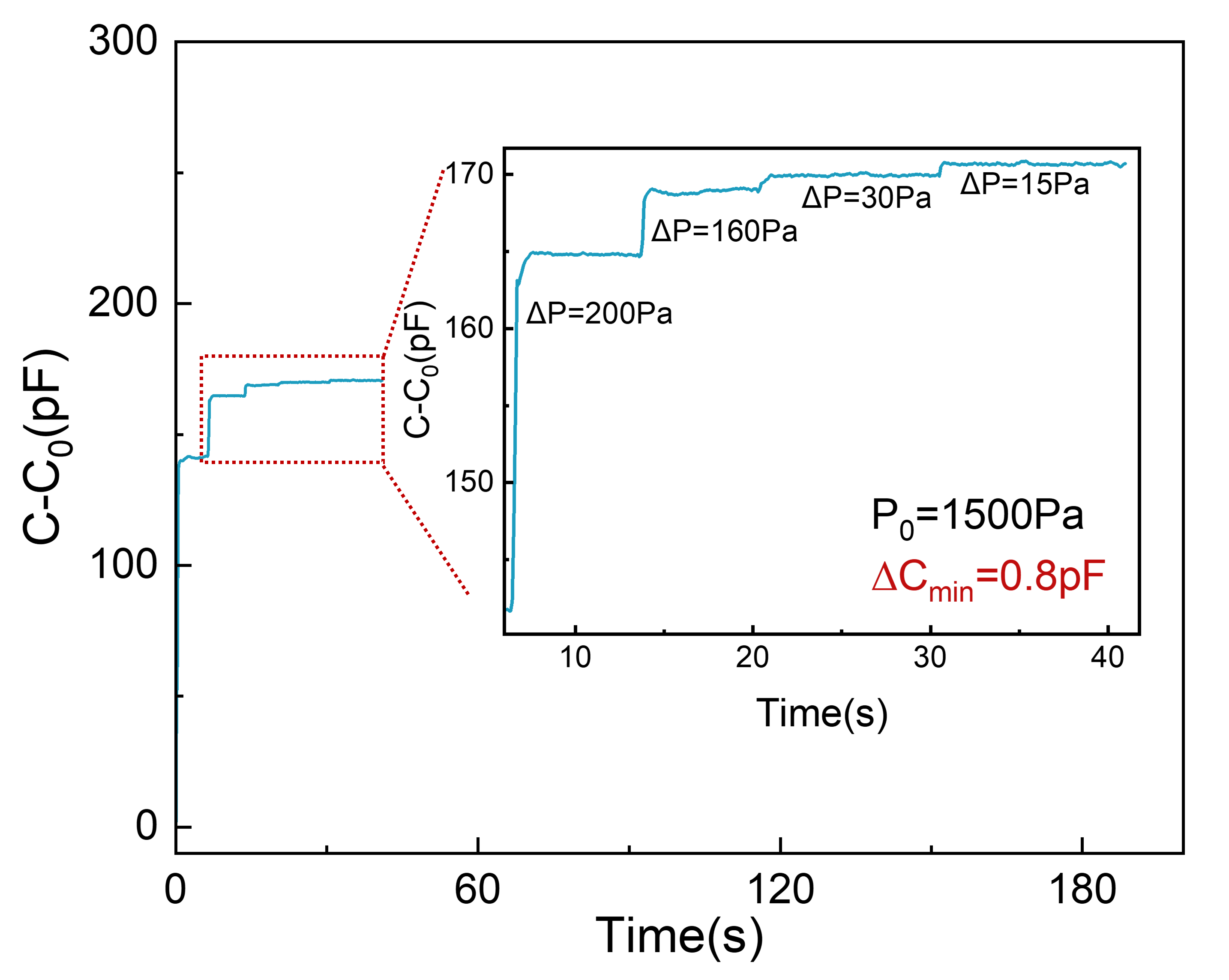


**Figure S12.** Detection of micro-pressure at a loading pressure of 1500 Pa.


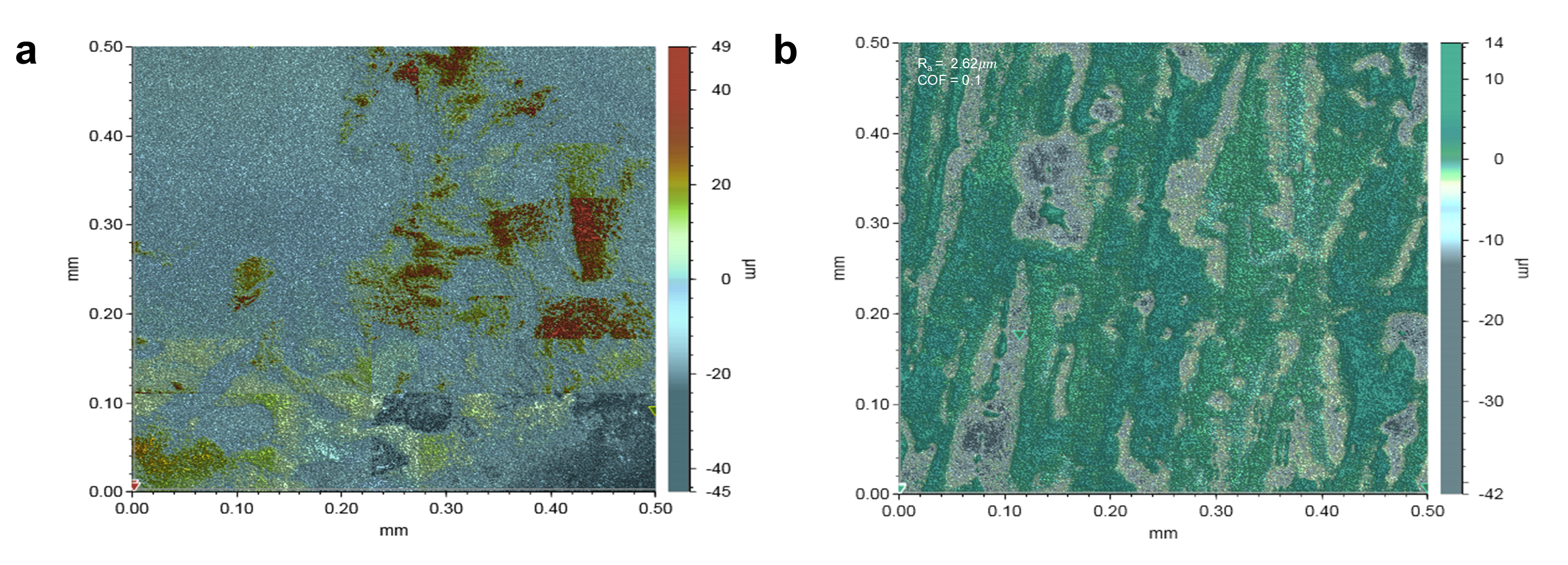


**Figure S13.** Laser confocal microscopy images showing the surface roughness of the electrode treated with 400 mesh (a) and treated with conductive lubricant (b).


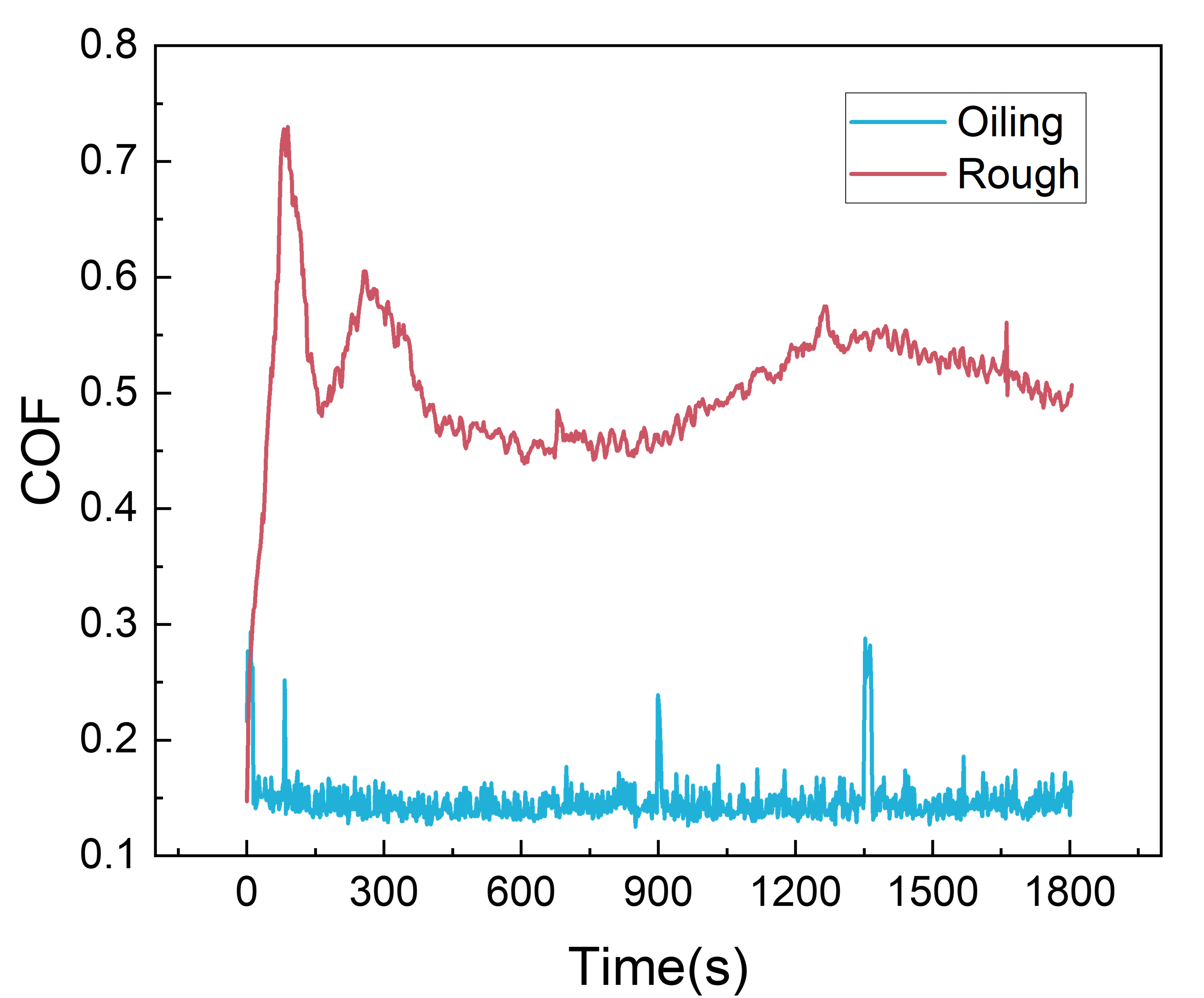


**Figure S14.** Coefficient of friction for lubricated electrodes and rough electrodes, tested with a friction stroke of 5 mm, a traveling speed of 50 mm/s, and linear reciprocating motion for 30 minutes at room temperature.


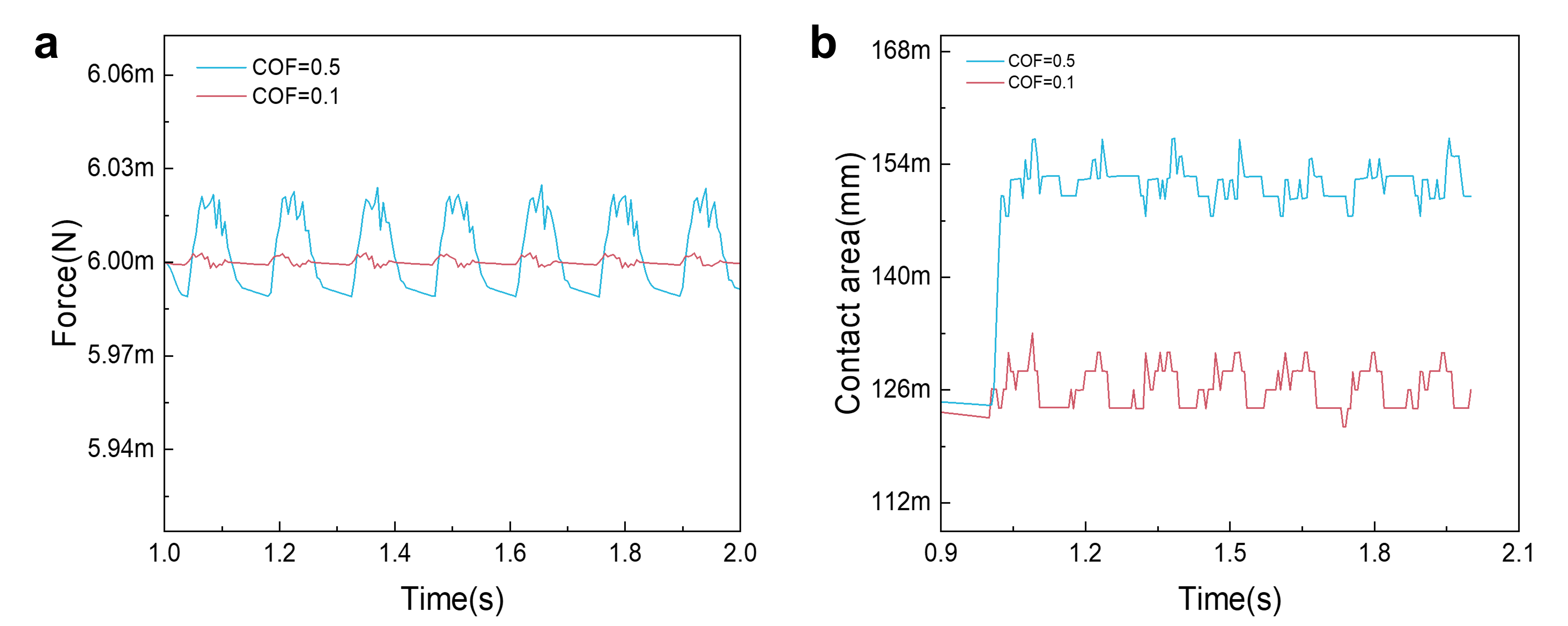


**Figure S15**. FEA stress (a) and contact area (b) of microstructures obtained by moving electrodes with different friction coefficients over the dielectric layer with 20% displacement. The results show that electrodes with small friction coefficients generate less normal force and contact area on the microstructured surface during movement.


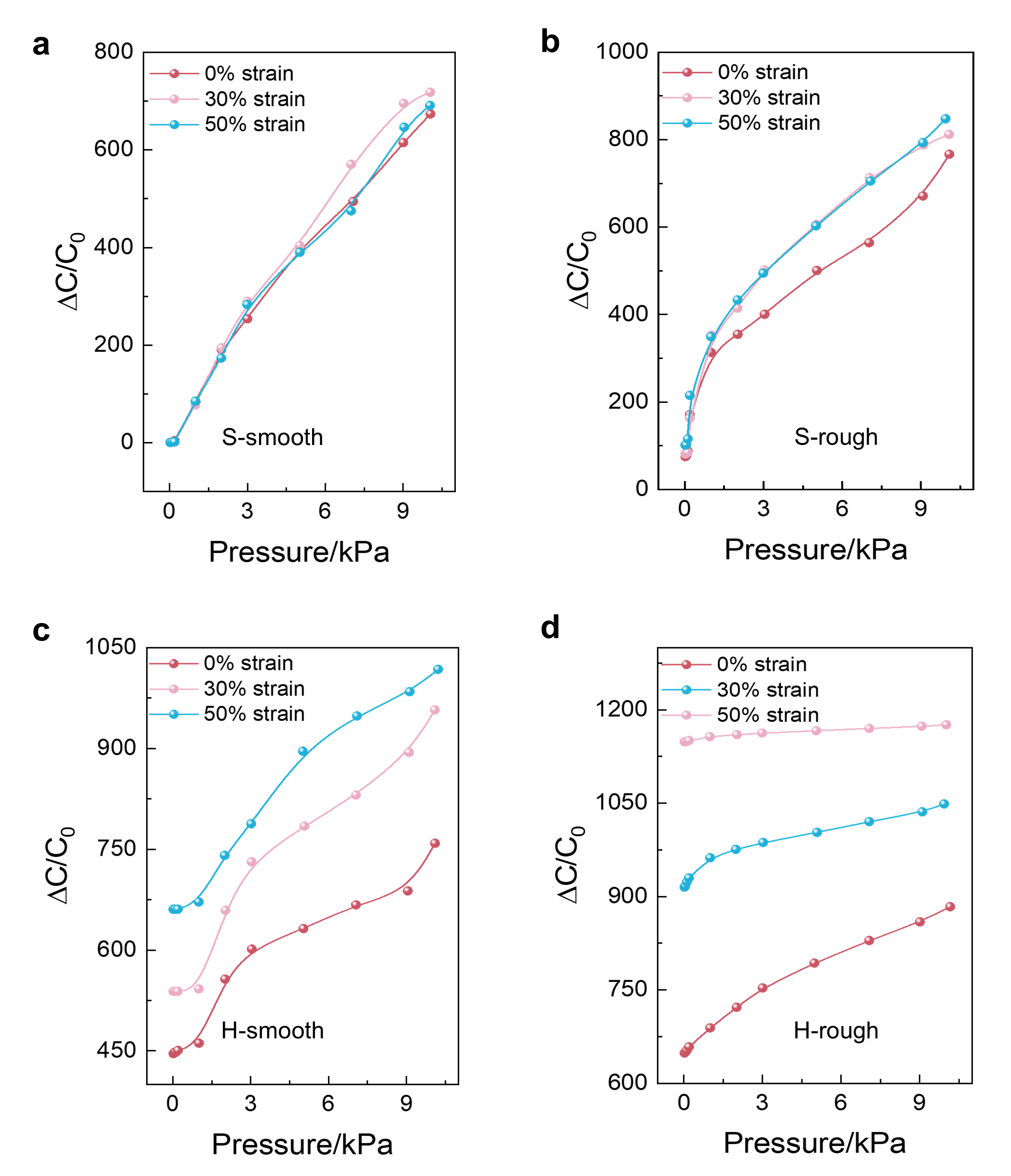


**Figure S16.** Normalized capacitive signal. (a) S-smooth, (b) S-rough, (c) H-smooth, (d) H-rough.


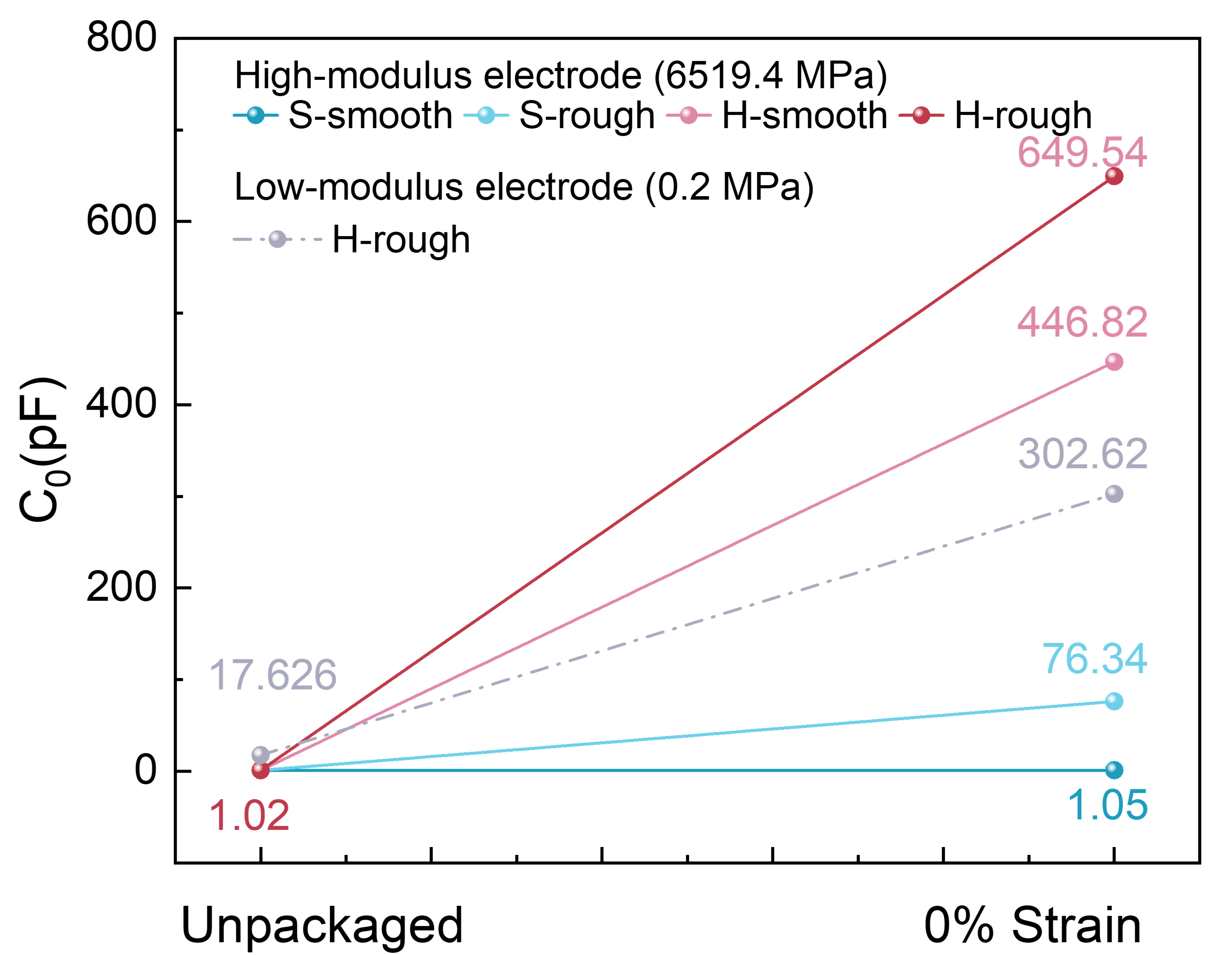


**Figure S17.** Initial capacitance of sensors with different package configurations.

The initial capacitance of sensors with different structures varies, mainly influenced by two factors: First, the incorporation of a fully PU hard-encapsulation structure increases pre-stress, thereby enhancing the initial capacitance, with the H-rough structure exhibiting a higher initial capacitance than the S-rough structure. Second, a higher friction coefficient between the electrode and dielectric layer results in a higher initial capacitance, thus the initial capacitance of the S-rough structure is greater than that of the S-smooth structure.


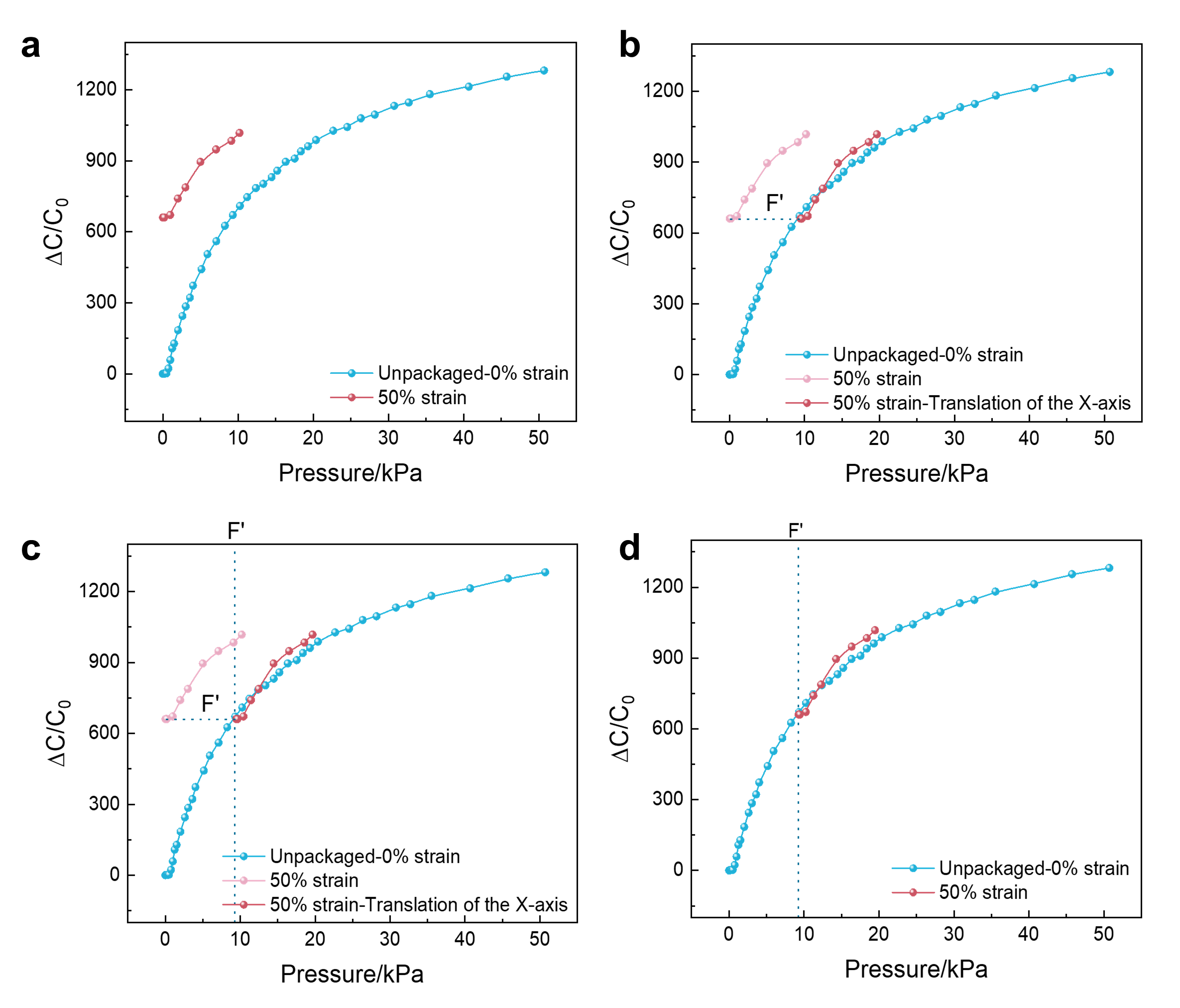


**Figure S18.** Data processing flowchart for calculating normal force (*F'*). (a) Normalized data of the encapsulated sensor at 50% stretch compared to the unencapsulated sensor curve. (b) Horizontal translation to align data with the unencapsulated curve, followed by (c) identifying the pressure F' at the initial ∆C/C_0_. (d) F' represents the pre-stress generated during the 50% stretch of the sensor.


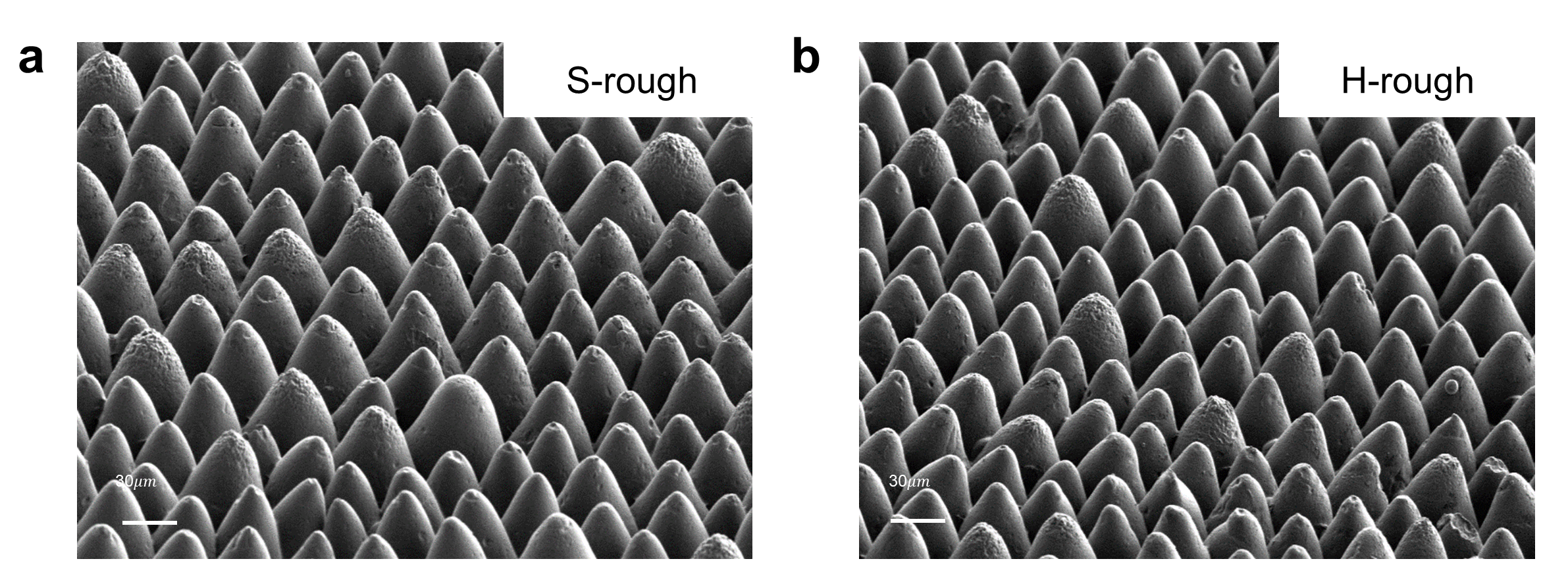


**Figure S19.** SEM images of the dielectric layers in S-rough (a) and H-rough (b) sensors after 50% strain stretching. The images show no significant damage at the microstructure tips of the dielectric layers, suggesting that the capacitance signal increases are due to enhanced interfacial friction rather than microstructure breakage.


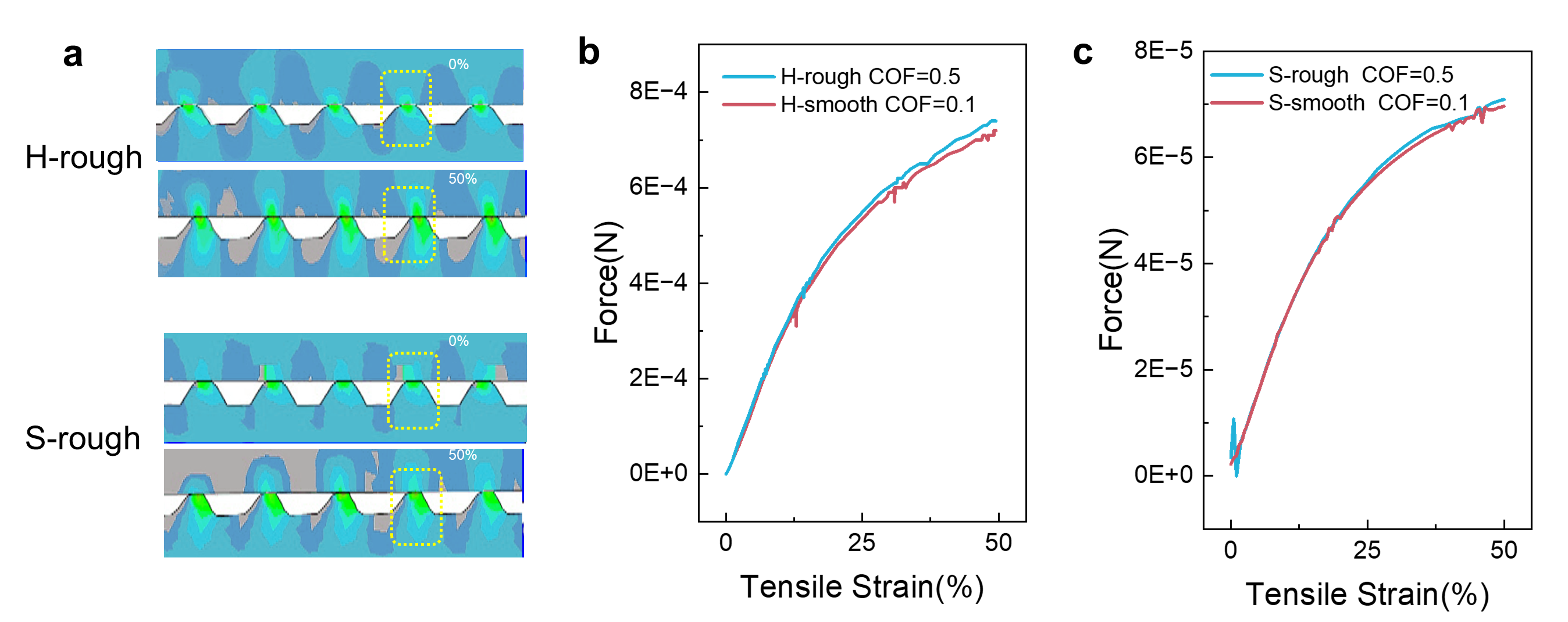


**Figure S20.** (a) FEA stress distributions of the H-rough and S-smooth sensor microstructures at 0% and 50% strain. (b, c) FEA stress plots showing the effect of the coefficient of friction using rigid encapsulation (b) and stretchable soft-hard encapsulation (c).


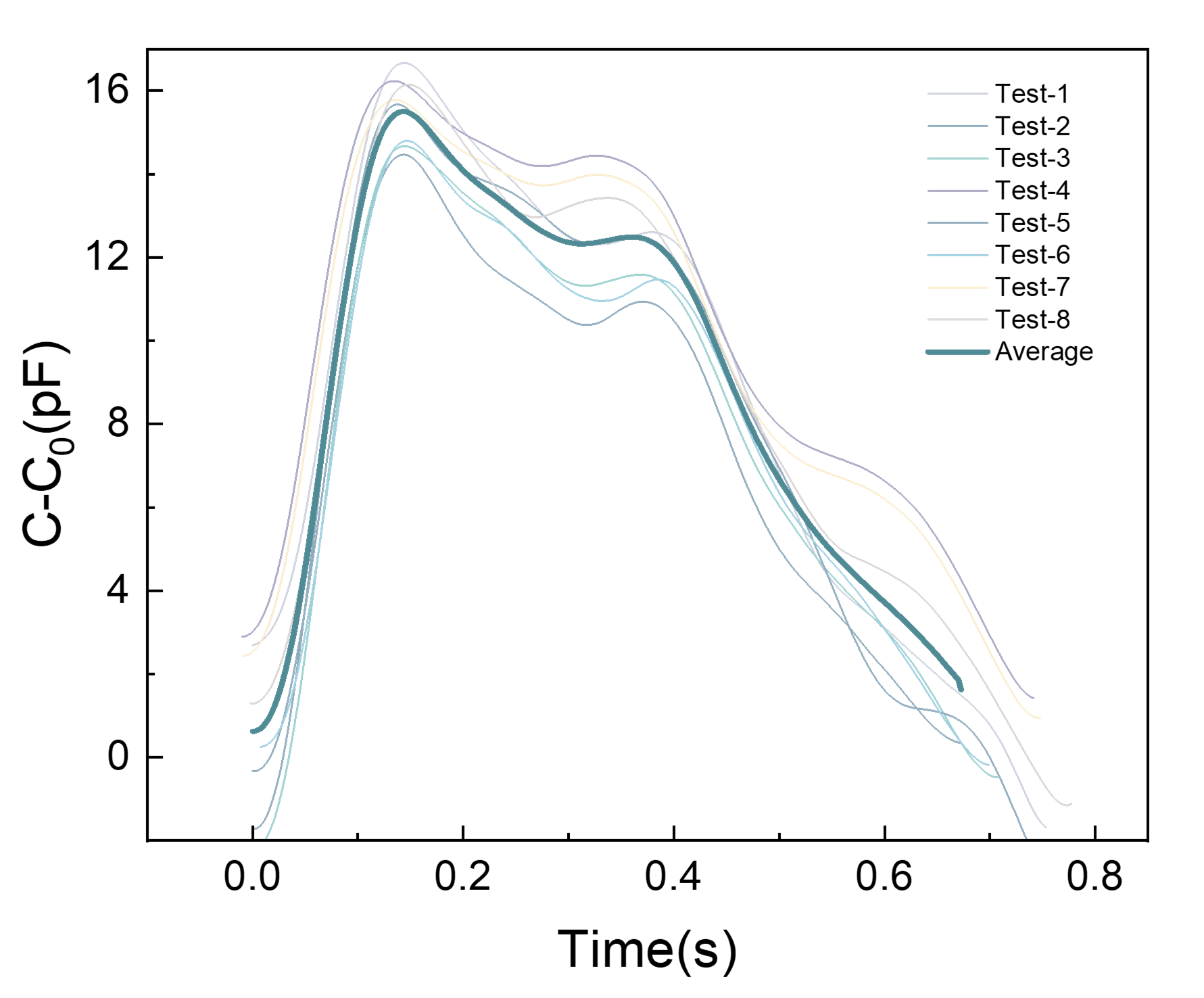


**Figure S21.** Fingertip pulse signal acquisition using the S-smooth sensor. Signals were obtained from the same tester every 24 hours, with 5s recordings taken over the course of one week.


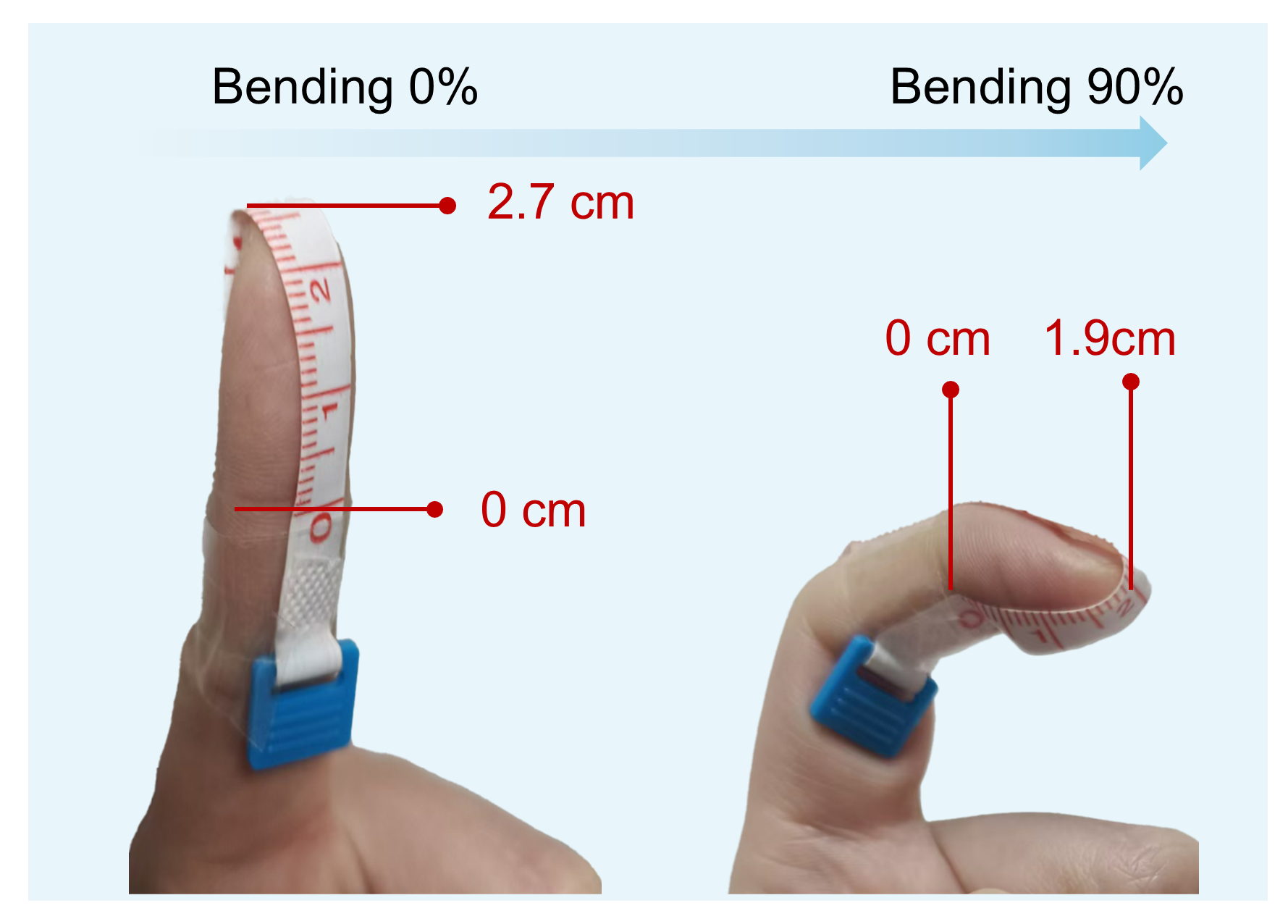


**Figure S22.** Strain calculation at 0° and 90° bending angles of the first phalanx.


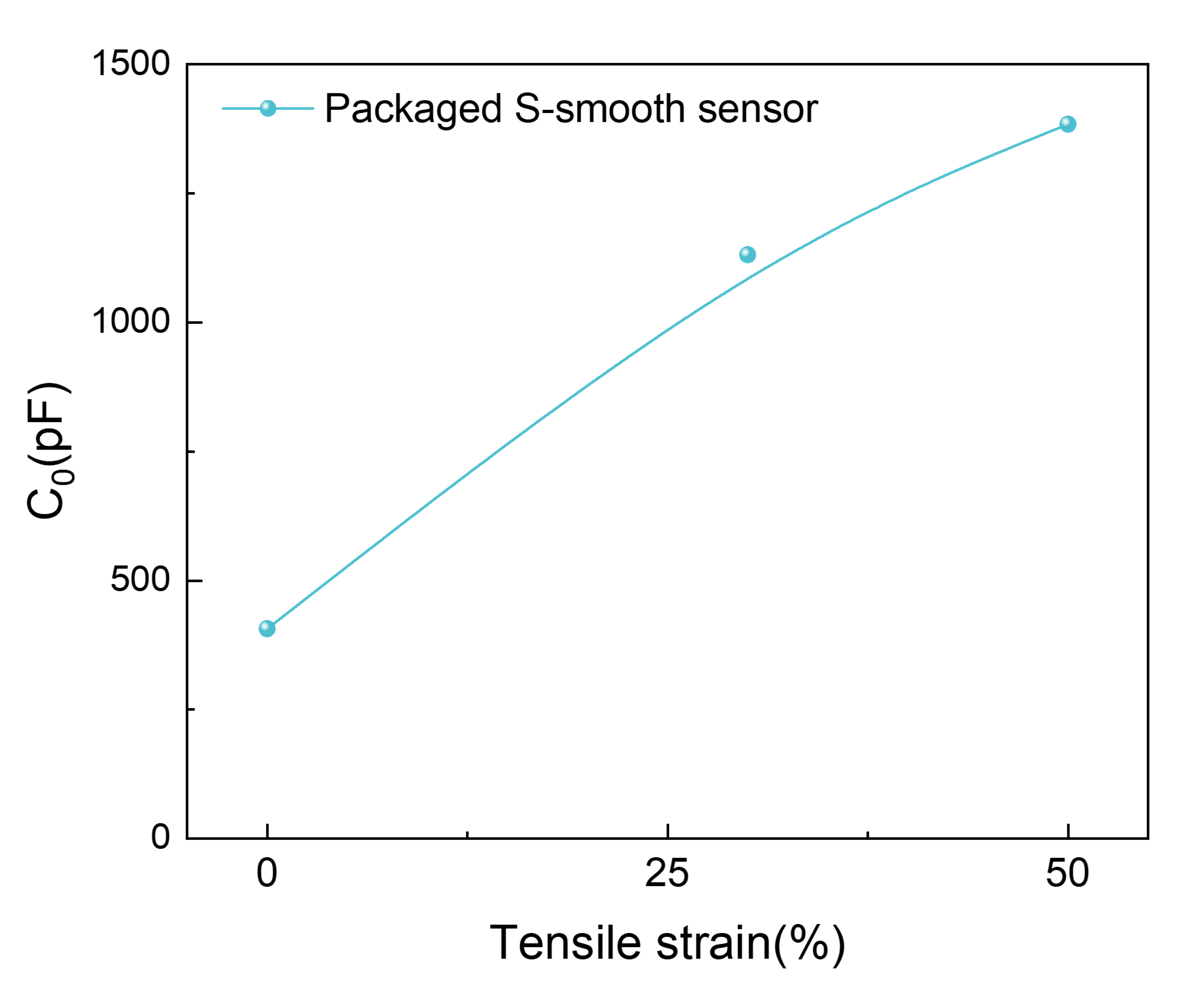


**Figure S23.** Initial capacitance signal fluctuations of the packaged S-smooth sensor under different strains conditions.


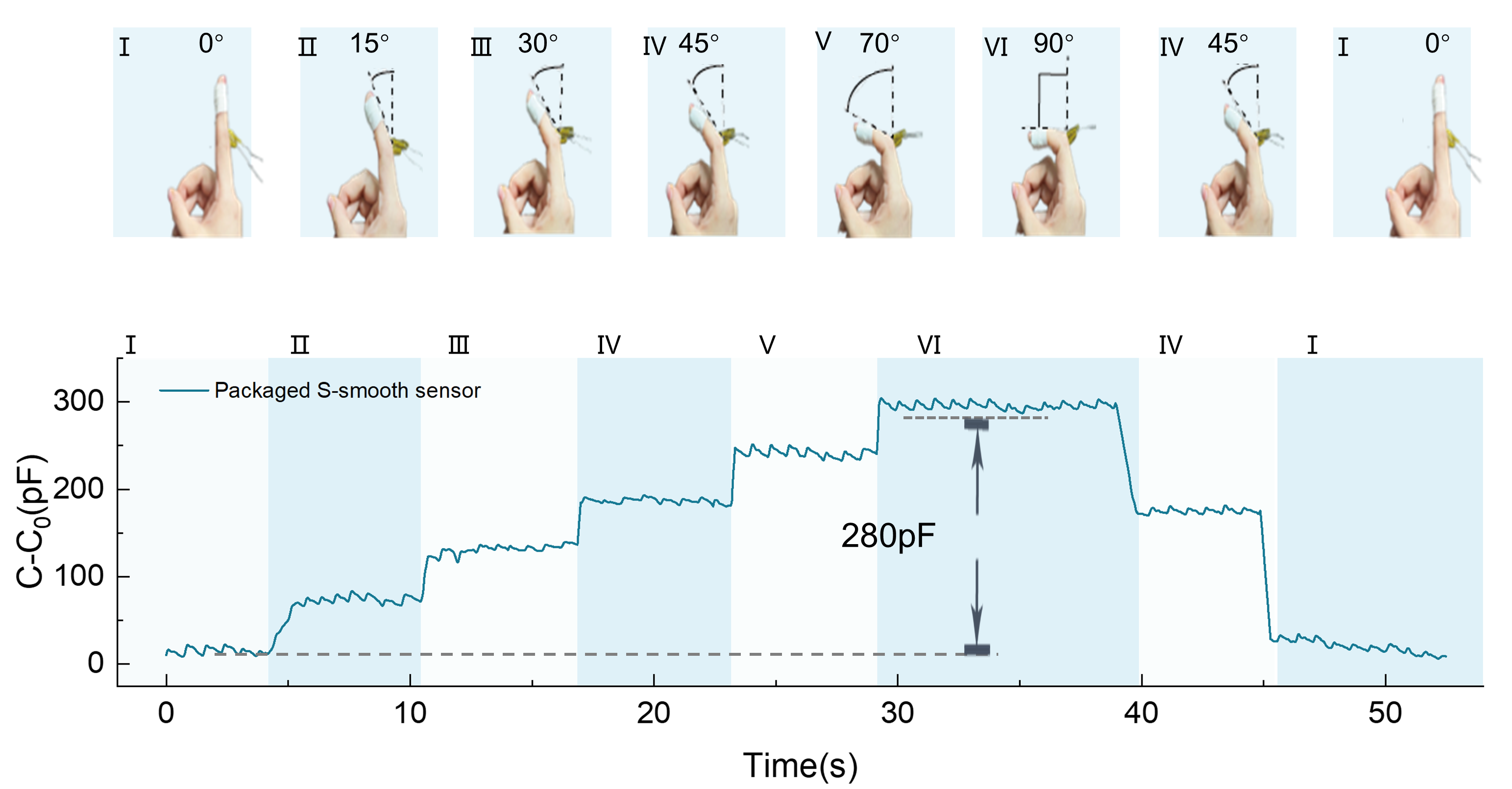


**Figure S24.** Fingertip pulse signals detected by the packaged S-smooth sensor at different finger bending angles.


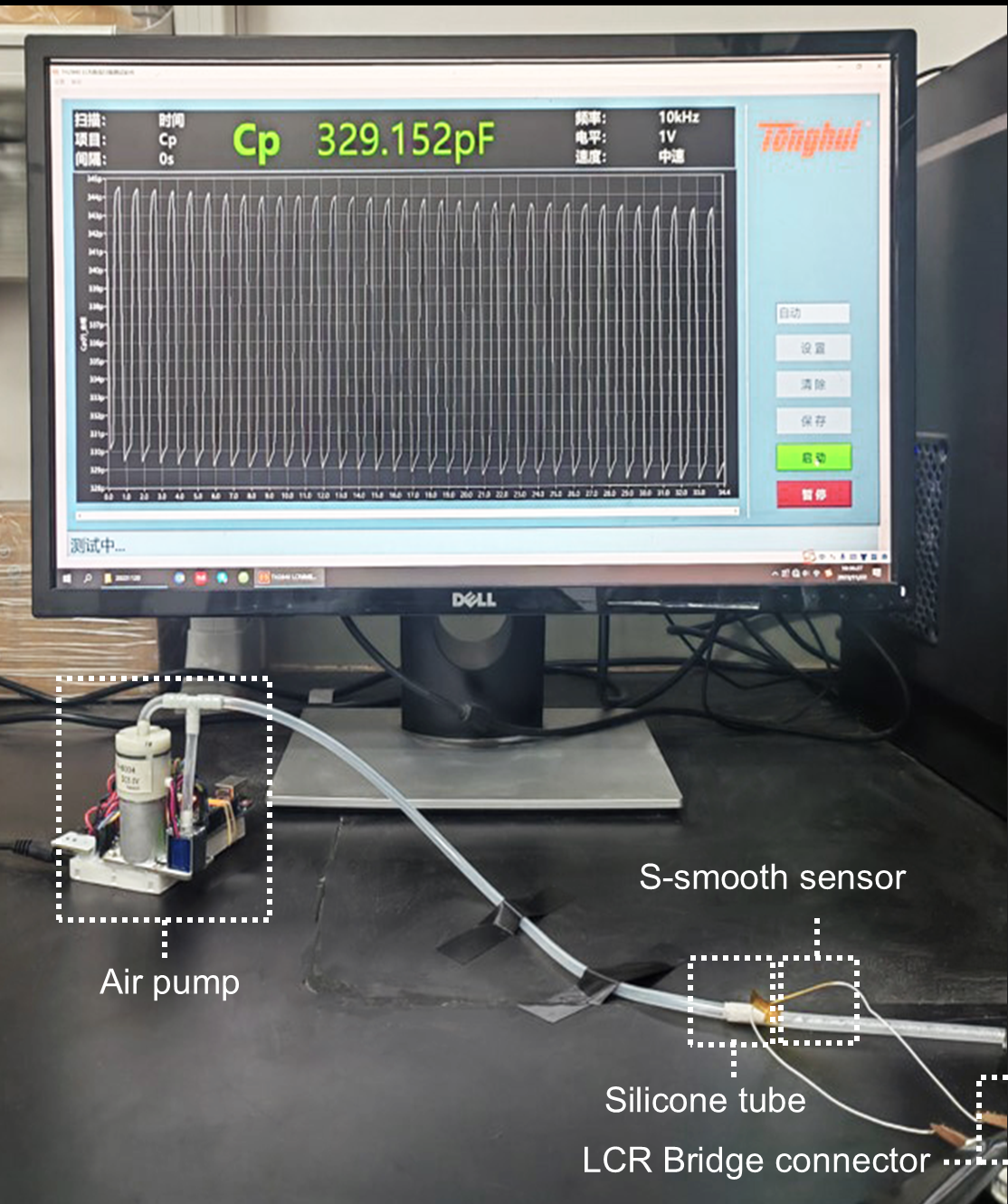


**Figure S25.** Digital image of the cyclic test setup for simulating pulse signals. The setup includes the capacitance signal acquisition interface, an air pump providing simulated vibration signals, and the S-smooth sensor attached to a 3 mm silicone tube that simulates a blood vessel.


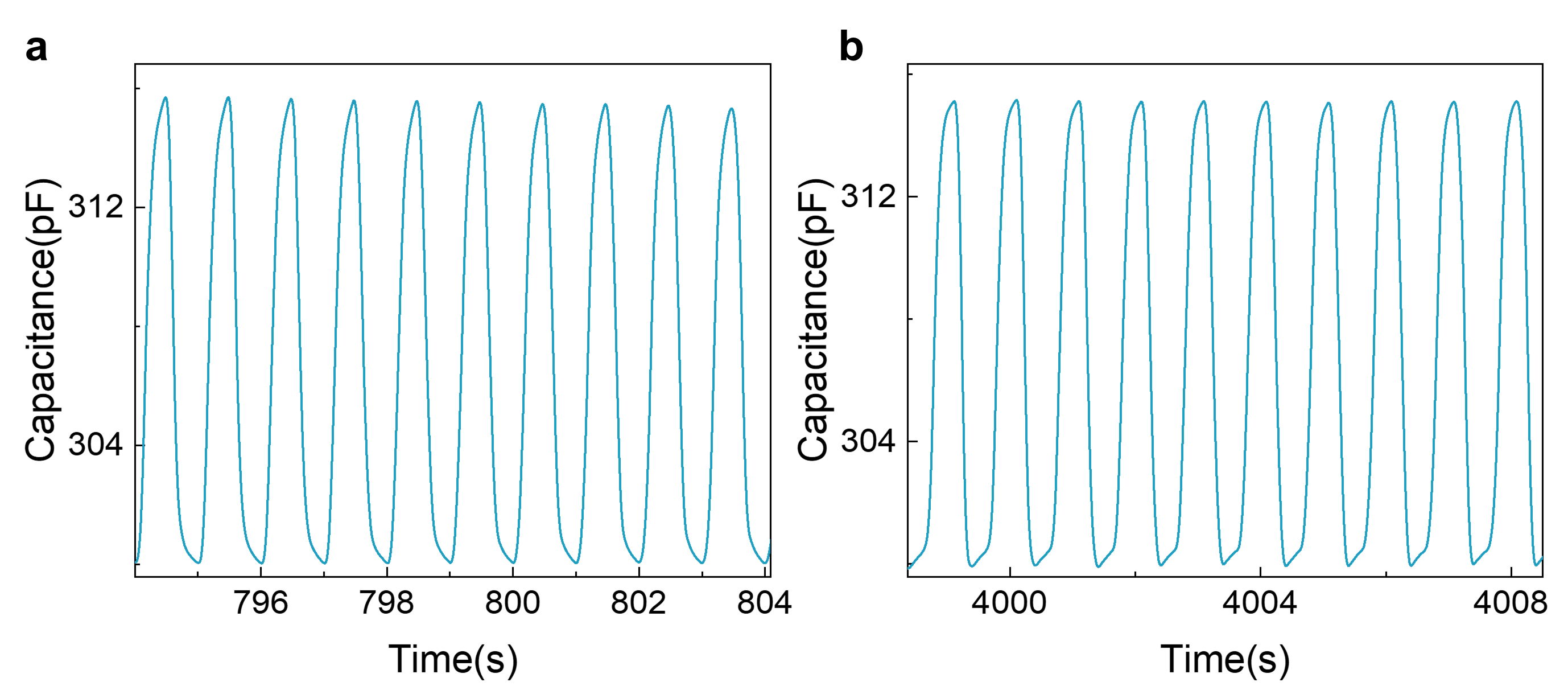


**Figure S26.** Capacitive signal after preload relaxation equilibrium during one million cycles. (a) Around 800 cycles, (b) After 4000 cycles.


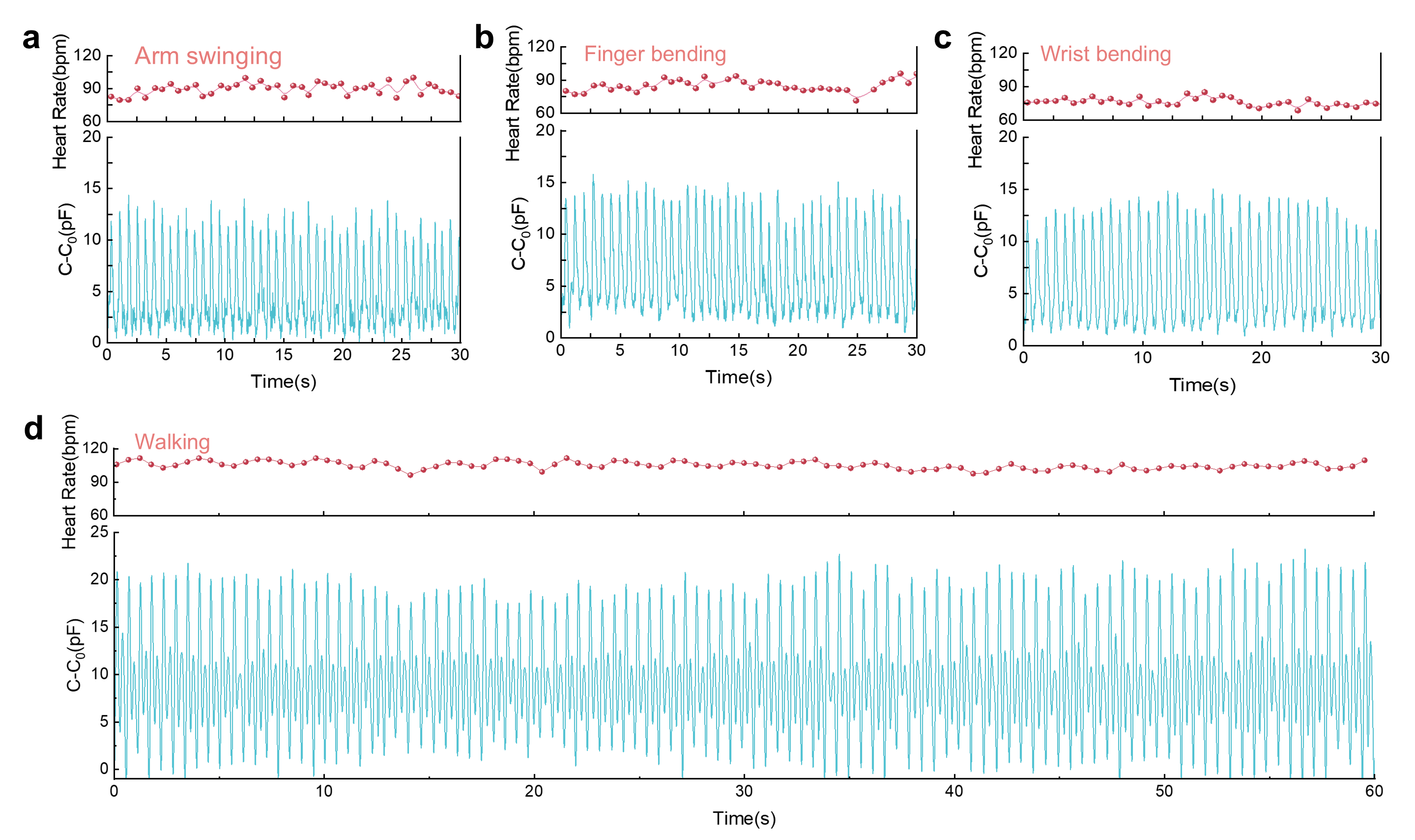


**Figure S27.** Capacitance changes of the S-smooth sensor for fingertip pulse detection under different movements, including arm swinging (a), finger bending (b), wrist bending (c), and walking (d). The dotted line plots show the extracted heart rate data for each movement.


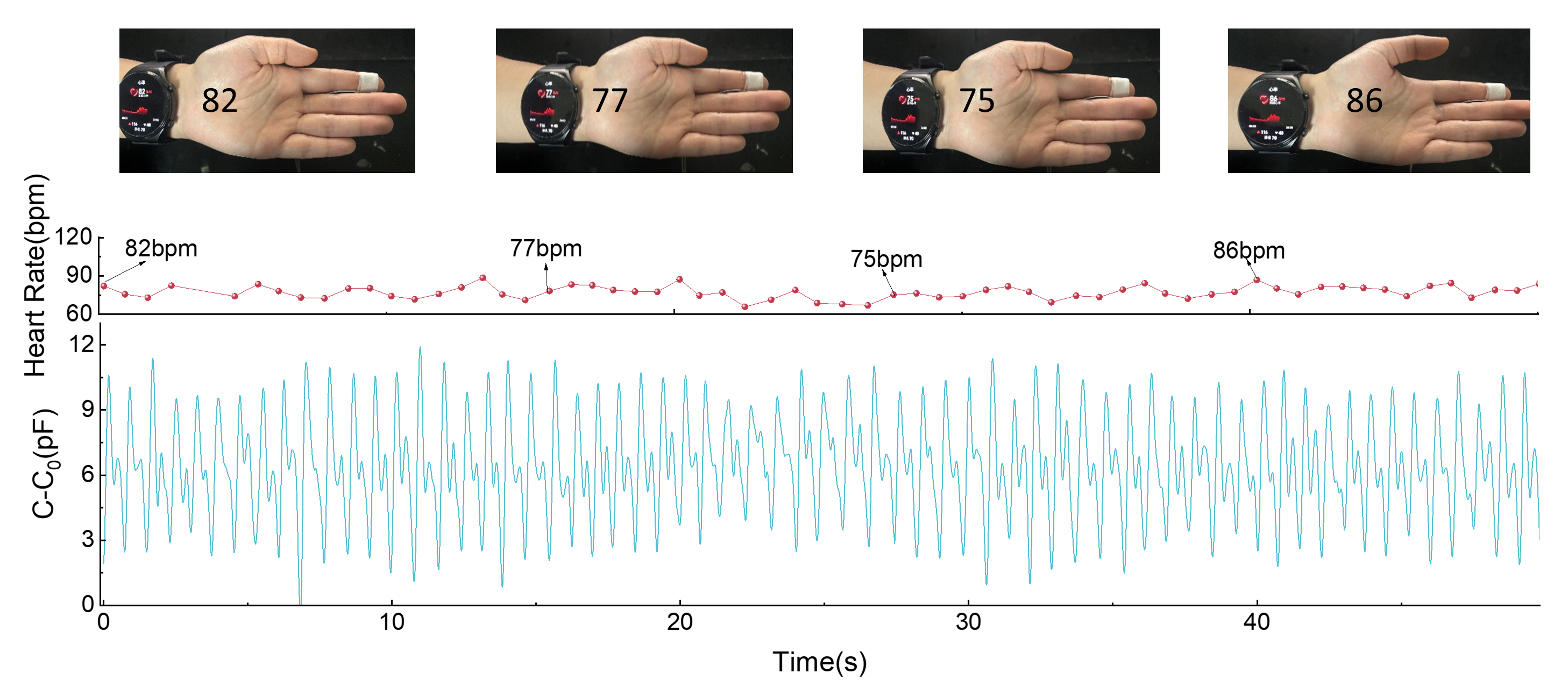


**Figure S28.** Heart rate detected by S-smooth sensors and commercial optoelectronic pulse detection device.
